# Functional characterization of diverse ring-hydroxylating oxygenases and induction of complex aromatic catabolic gene clusters in *Sphingobium* sp. PNB

**DOI:** 10.1016/j.fob.2014.03.001

**Published:** 2014-03-07

**Authors:** Pratick Khara, Madhumita Roy, Joydeep Chakraborty, Debajyoti Ghosal, Tapan K. Dutta

**Affiliations:** Department of Microbiology, Bose Institute, P-1/12 C.I.T. Scheme VII M, Kolkata 700054, India

**Keywords:** RHO, ring-hydroxylating oxygenase, ET, electron transport, PAHs, polycyclic aromatic hydrocarbons, ISP, iron-sulfur protein, GR, glutathione reductase, PDB, Protein Data Bank, IPTG, isopropyl-β-thiogalactopyranoside, NR, non-redundant, NBB, *n*-butylboronate, Sphingomonads, *Sphingobium* sp., Polycyclic aromatic hydrocarbons, Catabolic genes, Substrate specificities

## Abstract

*Sphingobium* sp. PNB, like other sphingomonads, has multiple ring-hydroxylating oxygenase (RHO) genes. Three different fosmid clones have been sequenced to identify the putative genes responsible for the degradation of various aromatics in this bacterial strain. Comparison of the map of the catabolic genes with that of different sphingomonads revealed a similar arrangement of gene clusters that harbors seven sets of RHO terminal components and a sole set of electron transport (ET) proteins. The presence of distinctly conserved amino acid residues in ferredoxin and *in silico* molecular docking analyses of ferredoxin with the well characterized terminal oxygenase components indicated the structural uniqueness of the ET component in sphingomonads. The predicted substrate specificities, derived from the phylogenetic relationship of each of the RHOs, were examined based on transformation of putative substrates and their structural homologs by the recombinant strains expressing each of the oxygenases and the sole set of available ET proteins. The RHO AhdA1bA2b was functionally characterized for the first time and was found to be capable of transforming ethylbenzene, propylbenzene, cumene, *p*-cymene and biphenyl, in addition to a number of polycyclic aromatic hydrocarbons. Overexpression of aromatic catabolic genes in strain PNB, revealed by real-time PCR analyses, is a way forward to understand the complex regulation of degradative genes in sphingomonads.

## Highlights

• Gene clusters responsible for aromatic degradation in *Sphingobium* sp. PNB were sequenced.
• The ferredoxin from sphingomonads is structurally unique.
• Substrate specificities of several ring-hydroxylating oxygenases were determined.
• Oxygenase capable of transforming alkylaromatics was characterized.
• Complex regulation of degradative genes was revealed by real-time PCR analyses.

## 1 Introduction

Bacteria of the genera *Sphingomonas, Novosphingobium*, *Sphingopyxis* and *Sphingobium,* commonly referred to as sphingomonads [1], are well known for their potential in bioremediation and industrial applications [2,3]. Sphingomonads are widespread in various aquatic and terrestrial environments and are isolated with an exceptionally high frequency, as compared to bacteria from other taxonomic groups [3]. The members of these genera are often isolated and studied because of their ability to degrade a wide range of recalcitrant natural and anthropogenic aromatic compounds, including polycyclic aromatic hydrocarbons (PAHs) [3]. Degradation pathways in sphingomonads and non-sphingomonads are quite similar but there is a low degree of homology between the genes/enzymes of the degradation pathways. The extraordinary metabolic diversity of sphingomonads is primarily due to the existence of multiple ring-hydroxylating oxygenases (RHOs) and the conservation of specific gene clusters. These bacteria supposedly evolved as independent group, restricting gene transfer to other bacteria and enabling these organisms to adapt faster to new potential carbon sources in the environment.

RHOs catalyze the initial oxidation step of a broad range of aromatic hydrocarbons including PAHs. RHOs have one or two soluble electron transport (ET) proteins, which deliver reducing equivalent to the α-subunit of the hetero-multimeric α_n_β_n_ or homo-multimeric α_n_ forms of terminal oxygenases for oxygen activation during catalysis [4]. Structural studies on representative oxygenases showed that the α-subunit of RHOs contains an N-terminal iron-sulfur protein (ISP) domain, with a conserved Rieske [2Fe–2S] center and a C-terminal catalytic domain having a conserved mononuclear iron-binding site [5]. RHO α-subunits have been classified on the basis of their evolutionary and functional behaviors, in relation to structural configuration of substrates and preferred oxygenation site(s) [6].

The sphingomonad strains *Sphingobium yanoikuyae* B1 [7], *Novosphingobium aromaticivorans* F199 [8], *Sphingobium* sp. P2 [9] and *Sphingomonas* sp. LH128 [10] are capable of degrading a wide range of aromatic compounds. All of them possess seven pairs of genes coding for the large and small subunits of RHOs and a single set of ET system, consisting of a ferredoxin and a ferredoxin reductase. The arrangement of degradative genes in sphingomonads is complex, with the genes scattered across several gene clusters, in contrast to the coordinately regulated organized operonic structure of genes in other bacteria. The metabolic versatility of sphingomonads is presumed to be due to their ability to oxidize a wide range of organic compounds, but the substrate specificities of the individual oxygenases are poorly studied. Furthermore, the regulation of the complex gene clusters in sphingomonads remains undefined.

*Sphingobium* sp. PNB isolated from municipal waste-contaminated soil is capable of growing with phenanthrene as the sole source of carbon and energy. Strain PNB can also utilize or co-metabolize a number of aromatic compounds including high-molecular weight PAHs [11,12]. The present study focuses on the molecular cloning, sequencing and organization of genes responsible for the degradation of various aromatic compounds, in order to understand the functional aspects of diverse RHO α-subunits. Further, the structural uniqueness of a single ferredoxin component involved in electron transfer to multiple RHO subunits and the induction profiles of the degradative genes in strain PNB were revealed, expanding the current perception of the complex catabolic architecture present in sphingomonads.

## 2 Results

### 2.1 Identification of RHO α-subunits in strain PNB

Based on the degenerate primers (Table S1), designed from multiple sequence alignment (MSA) using α-subunits of RHOs in sphingomonads, respective gene segments in strain PNB were amplified by PCR. Sequencing of the PCR products followed by blastx analyses confirmed the amplification of genes corresponding to α-subunits of six distinct RHOs, designated as *ahdA1b*, *ahdA1c*, *ahdA1d*, *ahdA1e*, *ahdA1f* and *xylX.* The primer designed to amplify *ahdA1a* failed to show any amplicon.

### 2.2 Cloning and sequence analysis of aromatic catabolic genes

**Fig. 1:**
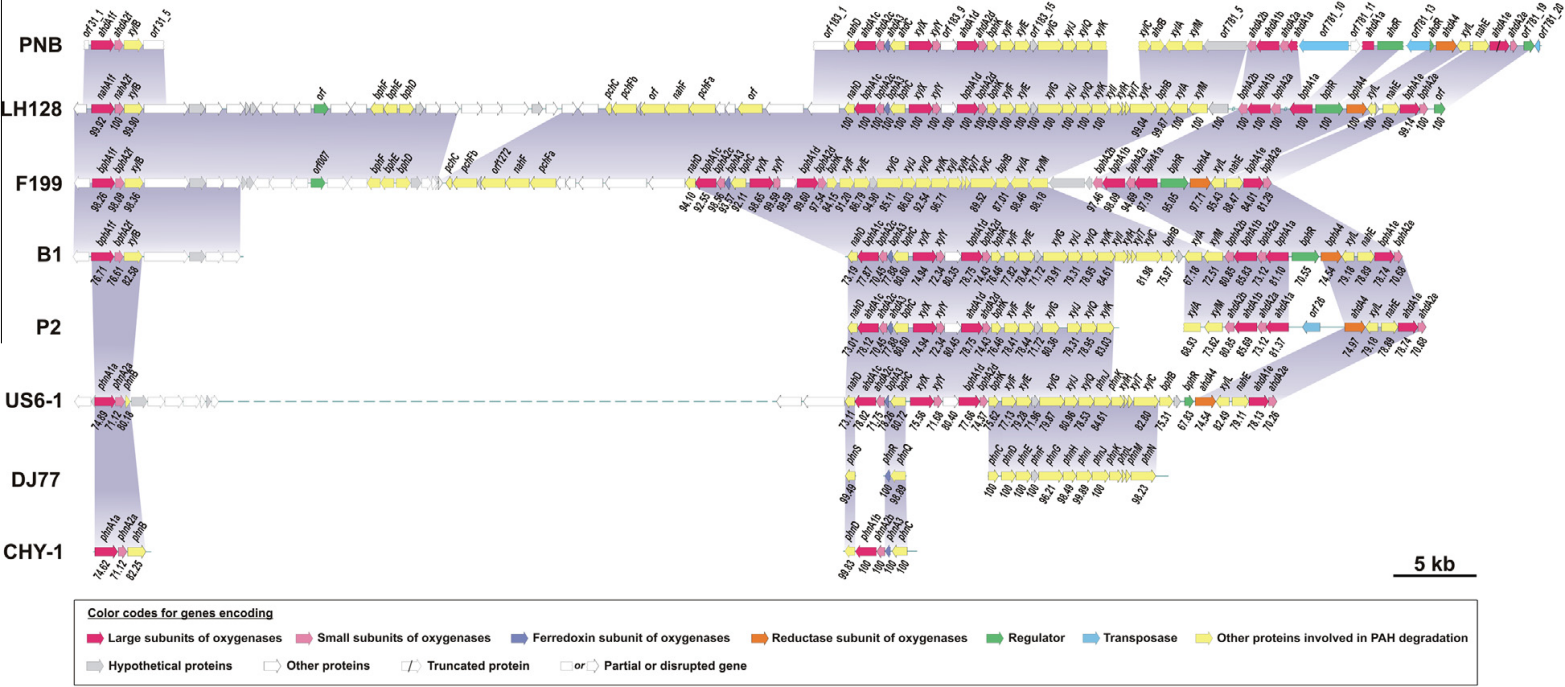
Mapping of aromatic hydrocarbon catabolic genes obtained from *Sphingobium* sp. PNB in comparison to the related catabolic genes in other sphingomonads. Numerical value below each gene indicates its sequence identity with the homologous gene in strain PNB. Shaded regions between the maps of a pair of organisms represent the locus of homologous gene segments. Dotted lines indicate presence of genes, which are not related to aromatic hydrocarbon catabolism.

Out of 1000 fosmid clones, eight supported desired PCR amplification with one and/or the other set of primer(s) corresponding to different RHO α-subunit genes (Table S1). Among them, fosmid clone FC-31 was found to harbor α-subunit gene specific for *ahdA1f*, clone FC-183 for *ahdA1c* and *ahdA1d* while clone FC-781 for *ahdA1b*, *ahdA1e* and *xylX*. Subcloning, screening based on the presence of RHO α-subunit gene(s) and sequencing, followed by sequence alignment and blast searches revealed the identification of putative ORFs. The subclones which did not serve as template for the amplification of RHO α-subunit gene(s), were also sequenced using M13 forward and reverse primers and analyzed. Those which showed the presence of putative genes involved in the metabolism of aromatic compounds were further sequenced by primer walking and analyzed as described above to identify additional putative genes and proteins of the degradative gene clusters. Further, gaps between genes expected to be in close proximity were bridged by a conventional primer walking method, using primers designed from the sequences at the proximal ends of the genes. Examination of sequence revealed 37 complete, 5 partial and 2 disrupted ORFs. Putative genes and proteins, identified from the above analyses are listed in Table S2. Based on protein sequence homology and conserved domain analyses, a number of genes are likely to be involved in PAHs or other aromatic degradation pathways. Sequenced aromatic catabolic gene clusters of strain PNB were mapped and compared with the homologous gene clusters reported in various sphingomonads (Fig. 1).

Aligned sequence data encode seven pairs of putative oxygenase α- and β-subunits (AhdA1_[a–f]_A2_[a–f]_, XylXY). Of which, the α-subunit, *ahdA1a* is disrupted by the insertion of a transposase and a resolvase, which prevented PCR amplification, as stated above. In addition, the α-subunit (*ahdA1e*) was found to be truncated, with a deletion of 10 nucleotides (between bases 646 and 647) with respect to the corresponding homologous genes in related sphingomonads [8,9,13]. Interestingly, this is the only RHO large subunit in sphingomonads with a consensus sequence of D-X-D-X_2_-H-X_4_-H, which is slightly different from that of the classical non-heme iron coordination site, E-X_3/4_-D-X_2_-H-X_4/5_-H [8,14].

The majority of the catabolic enzymes from strain PNB (Table S2) are 99-100% identical with those found in *Sphingomonas sp.* LH128 whole genome data (NCBI BioProject: PRJNA172017), while the rest showed maximum similarity to those encoded in either *Novosphingobium aromaticivorans* F199 [8] or *Sphingobium chungbukense* DJ77 [15]. The aromatic degradative genes identified in this study showed maximum identity to those found in other sphingomonads, viz. *Sphingomonas* sp. LH128, *Sphingobium yanoikuyae* B1, *Sphingobium* sp. P2, *Sphingomonas* sp. CHY-1 (described as the closest neighbor of genus *Sphingobium*) [16], *Sphingomonas chungbukensis* DJ77 (reclassified as *Sphingobium*), *Novosphingobium aromaticivorans* F199 but the 16S rRNA gene sequence (1451 bp) of strain PNB showed 92.11, 95.04, 95.17, 96.39, 95.36 and 92.85% identity, respectively. Observed sequence similarity at the catabolic gene level and 16S rRNA level along with the signatures of transposases found in the catabolic gene clusters of strain PNB as well as in other sphingomonads [3], clearly indicate gene transfer events during their evolution.

### 2.3 Phylogenetic analysis of multiple RHO α-subunits and ET protein sequences

**Fig. 2:**
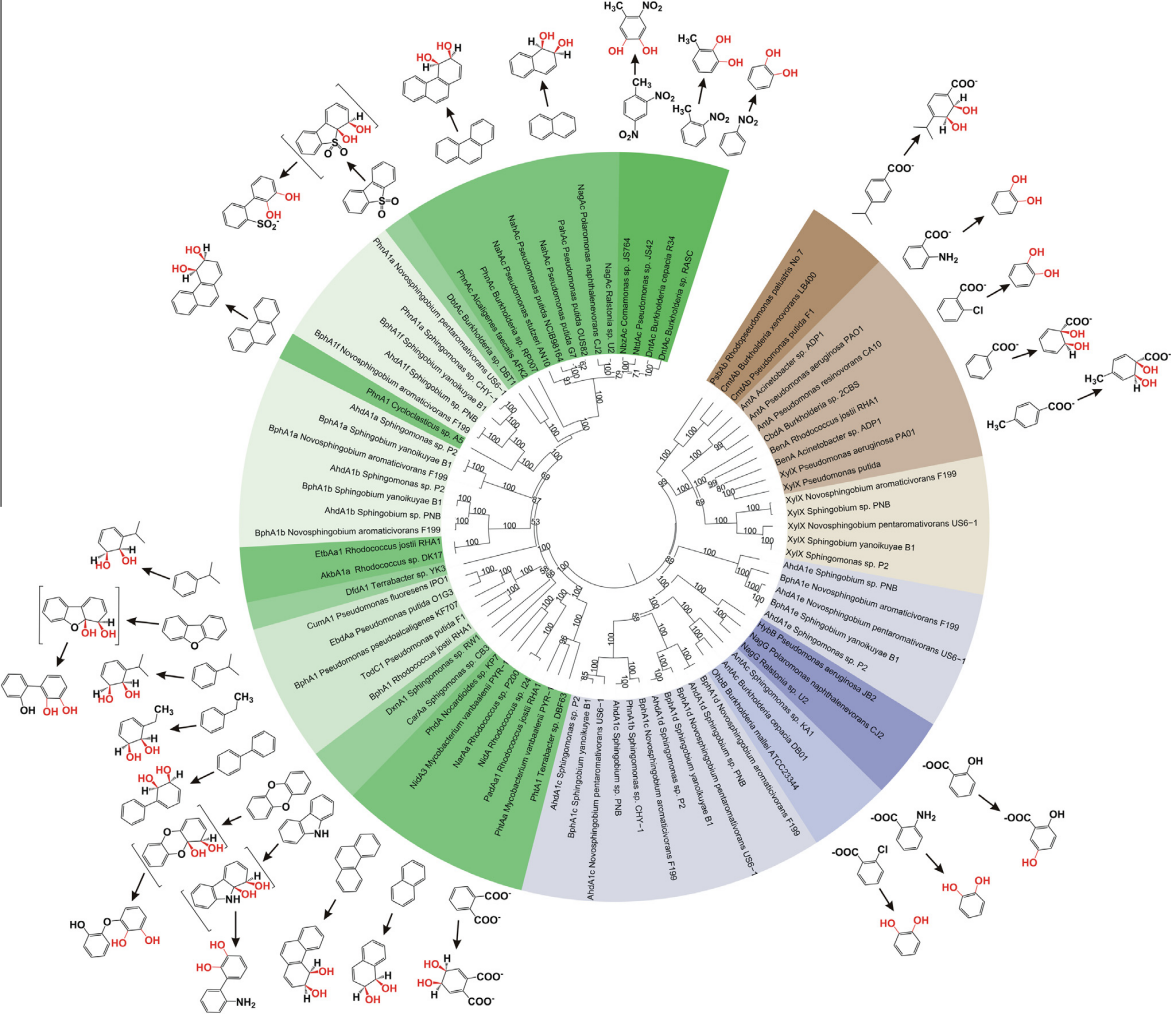
Dendogram showing the relatedness of α-subunit of bacterial aromatic RHOs along with the known biochemical reactions catalyzed. Class A, B and C RHOs (following classification scheme as described by Chakraborty *et al.*[6]) are shown in shades of green, brown and blue, respectively. Each shade within a class represents different reaction chemistry (with respect to oxygenation sites) while the lightest shade in each class represents the α-subunit belonging to sphingomonads. Values at each node indicate level of bootstrap support based on 100 resampled datasets while bootstrap values below 50% are not shown. A Class D carbazole dioxygenase (CarAaI) from *Sphingomonas* sp. KA1 (GenBank: YP_717981) was used as outgroup. (For interpretation of the references to colour in this figure legend, the reader is referred to the web version of this article.)

Sequence analysis revealed seven sets of putative α- and β-subunit RHO genes, with one of the α-subunits (*ahdA1a*) disrupted. Each α subunit contains an N-terminal ISP domain, with a conserved Rieske [2Fe–2S] center, a C-terminal catalytic domain having a conserved mononuclear iron-binding site and a conserved aspartate, which is known to facilitate inter-subunit electron transfer between ISP and catalytic domains of α-subunits [14,17,18]. The genes encoding β-subunits were found adjacent to that of the α-subunits in all the sets of oxygenases indicating possible co-evolution of α- and β-subunits and the presence of hetero-multimeric (α_n_β_n_)-type of RHOs in strain PNB. Fig. 2 illustrates the phylogenetic relation of the α-subunit protein sequences (AhdA1b, AhdA1c, AhdA1d, AhdA1e, AhdA1f and XylX) in strain PNB and the homologous sequences from other organisms, as mentioned in Table S3. Although the α-subunits in strain PNB share conserved domain regions, their nucleotide sequences and deduced amino acid sequences share limited homology with that of the nonsphingomonad counterparts. Moreover, phylogenetic analysis reveals that the individual α-subunit proteins in sphingomonads are distantly related (Fig. 2). Pairwise sequence alignments among the α-subunit in strain PNB, showed identities in the ranges of 55-64% and 25-48% at the levels of nucleotide and amino acid sequences, respectively. In previous studies, describing multiple RHOs in sphingomonads, substrate preferences of most of the RHOs have not been studied at length. Rather, the degradative genes have been annotated as *bph*, *phn* or *ahd* genes, merely on the basis of the aromatic compounds degraded by the individual species. A closer look at the phylogenetic tree of α-subunits reveals that the clustering depends broadly on substrate specificities [6]. Tree topology indicates that the homologous proteins first branch according to their substrate class preferences and within each branch, more similar sequences group in accordance with substrate sub-classes and ultimately, cluster according to the species tree. It has been observed that each of the homologous α-subunit proteins from sphingomonads clusters together in different clades (Fig. 2). Apart from the homologous α-subunit proteins from sphingomonads (78–100% identity), few homologous α-subunits were also detected from the whole genome sequence of *Cycloclasticus* sp. P1, which showed up to 64% sequence identity to the α-subunit proteins determined in strain PNB. According to the classification suggested by Chakraborty *et al.* [6], AhdA1b and AhdA1f of strain PNB and their homologues belong to A-IIIαβ type RHOs where AhdA1f corresponds to well studied PAH dioxygenases in sphingomonads [7,10,19] while AhdA1b correspond to ethylbenzene dioxygenase (72.8% identity) in *Rhodococcus jostii* RHA1 [20], one of the least explored A-IIIαβ type RHOs. On the other hand, XylX has largely been described as benzoate/toluate dioxygenase belonging to B-IIαβ type RHO [8]. The α-subunit from strain PNB, designated as *xylX*, also clustered with benzoate/toluate dioxygenases present in various genera and showed maximum identity (50.8%) with the well characterized benzoate dioxygenase from *Pseudomonas putida* [21]. On the other hand, AhdA1c, AhdA1d, and AhdA1e, all of which belong to C-IVαβ type RHOs, branched into three different subclusters (Fig. 2). AhdA1c and AhdA1d showed 49.75 and 47.25% identity with biochemically characterized *o*-halobenzoate dioxygenases of *Achromobacter xylosoxidans* A8 [22] and *Burkholderia mallei* ATCC 23344 [23] respectively. Similarly, AhdA1e clustered distinctly along with that of the homologous α-subunits from other sphingomonads and shared a common ancestry with *o*-halobenzoate dioxygenase and salicylate 5-hydroxylase.

**Fig. 3:**
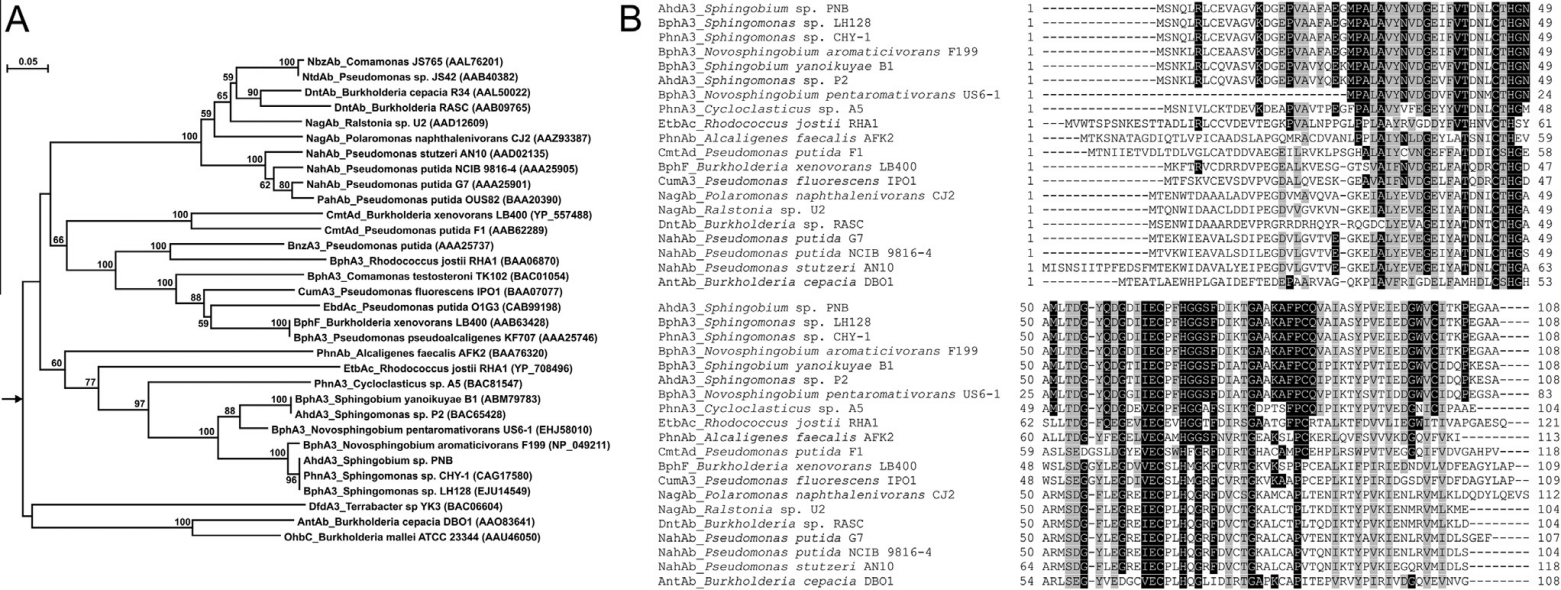
(A) Neighbor-joining tree and (B) sequence alignment of Rieske type [2Fe–2S] ferredoxins from different well studied xenobiotic degrading bacteria. In the tree, values at each node indicate level of bootstrap support based on 100 resampled datasets while bootstrap values below 50% are not shown. An unrelated ferredoxin (PhtAc) from *Mycobacterium vanbaalenii* PYR-1 (GenBank: AAQ91918) was used as outgroup and position of the root has been indicated by an arrow. Bar represents 0.05 substitutions per amino acid. Identical and similar residues in the sequence alignment are shaded in black and grey, respectively.

As in other sphingomonads, only one ferredoxin and one ferredoxin reductase were identified, each encoded in a separate cluster [3]. Analysis of AhdA3 revealed it to be a Rieske [2Fe–2S] type ferredoxin with the conserved C-X-H-X_n_-C-X_2_-H motif, a distinct feature of this family [24]. Fig. 3A illustrates the phylogenetic relation of AhdA3 with those of homologous sequences from various xenobiotic degrading organisms described in Table S4. Although these proteins share a conserved domain, AhdA3 shares limited sequence homology (<50% identity) with its non-sphingomonad counterparts. It is evident from the dendogram that the ferredoxins of the sphingomonads are highly similar (81–100% identity at amino acid level) and cluster together. It may be mentioned that AhdA3 and homologous sequences in sphingomonads displayed a few differentially conserved amino acids across the length of the proteins (Met26, Asn33, Gln57, Ile61, Phe66, Gly68, Ser70, Ala77, Ala80 and Phe81) in comparison to that of non-sphingomonads (Fig. 3B). However, the ferredoxins from few non-sphingomonads, viz. EtbAc, PhnA3 and PhnAb, complementing ethylbenzene dioxygenase in *Rhodococcus jostii* RHA1, PAH dioxygenase in *Cycloclasticus* sp. A5 and phenanthrene dioxygenase in *Alcaligenes faecalis* AFK2, respectively, were found to be phylogenetically close to the sphingomonad ferredoxins with significant sequence similarity (Fig. 3). This observation was in congruence with the phylogenetic relatedness of corresponding oxygenase from these organisms to those of the sphingomonads (Fig. 2), indicating a possible event of lateral transfer of oxygenase gene clusters among them.

The ferredoxin reductase encoded by *ahd*A4 belongs to glutathione reductase (GR)-type. It showed a maximum of 40.11% identity with the biochemically characterized ferredoxin–NAD^+^ reductase components of ethylbenzene dioxygenase present in nonsphingomonad strain *Rhodococcus jostii* RHA1 [20]. Fig. S1 shows the phylogenetic relationship of AhdA4 with the homologous sequences from various xenobiotic degrading organisms listed in Table S5. It has been observed that ferredoxin reductase sequences from sphingomonads cluster together (72–100% identity at amino acid level), similar to that obtained with the corresponding terminal oxygenase and ferredoxin components.

### 2.4 Homology modeling of terminal oxygenase subunits and ferredoxin proteins

Secondary structure prediction of the translated protein sequence of the terminal oxygenase α- and β-subunits (AhdA1fA2f) revealed that both proteins belong to structural class ‘alpha and beta’ proteins (a+b) (SCOP: 53931) whereas the ferredoxin (AhdA3) belongs to ‘all beta’ class (SCOP: 48724) with three stacked beta sheets. A search for homologs of the above proteins in the Brookhaven Protein Data Bank (PDB) yielded a close resemblance with that of the oxygenase components (PDB: 2GBX:A; 2GBX:B) and ferredoxin (PDB: 2I7F:A), respectively of the biphenyl 2,3-dioxygenase from *Sphingobium yanoikuyae* B1. AhdA1f showed 78.6% identity over 454 amino acids whereas AhdA2f showed 68.39% identity over 174 amino acids with 2GBX:A and 2GBX:B, respectively. On the other hand, AhdA3 was found to be 81.48% identical over 108 amino acids with 2I7F:A. Using these chains as templates, models of the terminal oxygenase α and β-subunits (AHD-O_PNB_) and ferredoxin (AHD-F_PNB_) proteins were generated. Qualities of the modeled structures were found to be satisfactory, as observed from PROCHEK, VERIFY3D and VADAR analyses.

### 2.5 Molecular docking of oxygenase–ferredoxin complexes

**Fig. 4:**
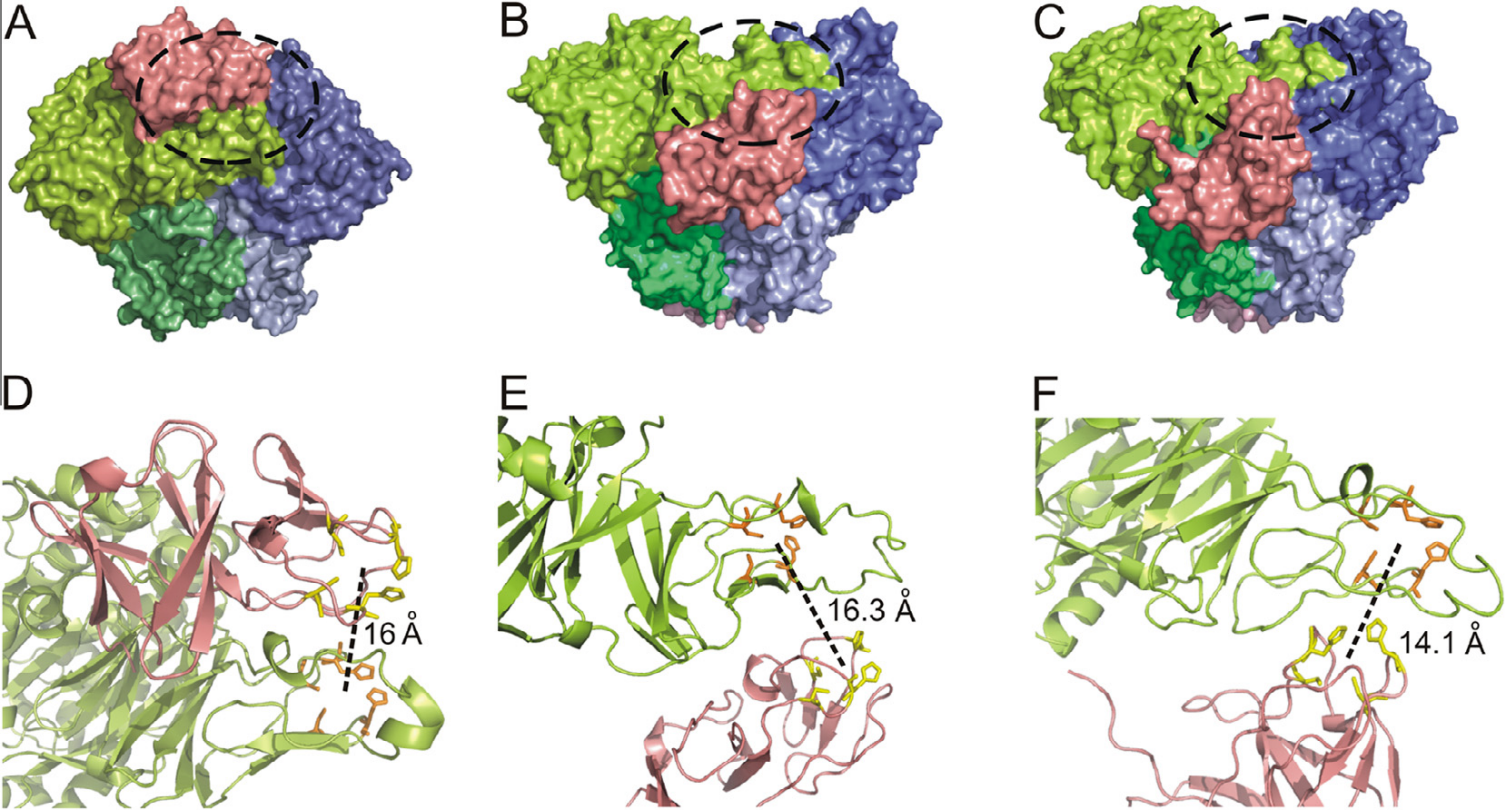
Molecular docking of oxygenase–ferredoxin complexes. The surface plots (side view) of the docked complexes of respective ferredoxin and terminal oxygenase components of (A) naphthalene 1,2-dioxygenase from *Pseudomonas putida* NCIB 9816-4, (B) biphenyl 2,3-dioxygenase from *Sphingobium yanoikuyae* B1 and (C) terminal oxygenase AhdA1fA2f from *Sphingobium* sp. PNB. In each structure, the visible α-subunits are colored in light green and blue, while the visible β-subunits are shown in dark green and slate. The ferredoxins in each complex are colored pink. Black dotted circle in each complex shows the region where the Rieske clusters of ferredoxin and oxygenase large subunit lie in close proximity for electron transport, while the same as enlarged (D, E and F) are shown in the corresponding cartoon representations. Distance between each pair of redox centre is shown in black dotted lines. (For interpretation of the references to colour in this figure legend, the reader is referred to the web version of this article.)

Docking experiments were performed using GRAMM-X to assess the interactions between three different oxygenase–ferredoxin complexes, viz. NDO-O_98164_:NDO-F_98164_, BDO-O_B1_:BDO-F_B1_ and AHD-O_PNB_:AHD-F_PNB_. Out of 50 docked complex models, ranked according to the scoring function, one showing distance between the Rieske clusters of ferredoxin and oxygenase α-subunit closest to 14 Å threshold [25] was considered as the best-fit model (Fig. 4). The observed distances were 16, 16.3 and 14.1 Å respectively for NDO-O_98164_:NDO-F_98164_, BDO-O_B1_:BDO-F_B1_ and AHD-O_PNB_:AHD-F_PNB_ complexes. For each docked complex, the interface residues of both ferredoxin and oxygenase α-subunit are shown in Table S6. As observed from the docked poses, NDO-F_98164_ binds at the depression between two adjacent α-subunits of NDO-O_98164_, which is in congruence with an earlier study [26]. On the contrary, both BDO-F_B1_ and AHD-F_PNB_ seem to bind at a pronounced depression formed by two α- and two β-subunits (Fig. 4), the other putative ferredoxin binding site, as postulated by Ashikawa *et al.* [26]. Mapping of predicted interface residues onto the alignment of ferredoxin sequences obtained from various xenobiotic degrading organisms depicted a few differentially conserved amino acid residues (Phe66, Gly68 and Phe81) in sphingomonads (Fig. 3B). Thus, it is believed that the Rieske-type [2Fe–2S] ferredoxins of sphingomonads might have evolved to complement multiple oxygenases present in these organisms.

### 2.6 Functional expression of RHOs

In order to investigate the substrate specificities of the six RHOs present in strain PNB, corresponding α-subunits along with the evolutionarily-related β-subunits were PCR-amplified and cloned into pET28a. To provide the terminal oxygenase component with an appropriate ET system, *ahdA3* (ferredoxin) and *ahdA4* (ferredoxin reductase) genes were cloned in a pET28a compatible vector, pCDF-1b. The construct harboring ET components were co-transformed individually along with each of the constructs of terminal oxygenases into *Escherichia coli* (*E. coli*) BL21(DE3). The recombinant *E. coli* strains when induced with isopropyl-β-thiogalactopyranoside (IPTG), produced appreciable levels of characteristic polypeptides, indicative of the expression of various components of oxygenases including ET proteins as revealed by SDS-PAGE analysis (data not shown). The recombinant *E. coli* strains producing multi-component oxygenases were incubated overnight separately with several aromatics based on their phylogenetic affiliation to substrate classes. GC–MS analyses of the *n*-butyl boronated (NBB) derivatives of the neutral extract of the water-soluble products released into the culture medium indicated the formation of aromatic dihydrodiols (Table 1). Among the oxygenases, AhdA1fA2f, belonging to A-IIIαβ sub-class of RHOs, was shown to dioxygenate naphthalene, phenanthrene, anthracene, biphenyl, acenaphthene, benzo[a]pyrene and benz[a]anthracene to the respective dihydrodiols. While AhdA1bA2b, phylogenetically belonging to same sub-class (A-IIIαβ) of RHOs (although, AhdA1b displayed an identity of 39% at the amino acid level with AhdA1f), could also dioxygenate naphthalene, phenanthrene, anthracene, biphenyl, acenaphthene, benzo[a]pyrene, benz[a]anthracene to their respective dihydrodiols, apart from dioxygenating ethylbenzene, propylbenzene, cumene and *p*-cymene. Recombinant strains, expressing AhdA1fA2f and AhdA1bA2b individually along with the same set of ET proteins were also found to transform indole to indigo. However, in comparison to AhdA1fA2f, transformation of indole to indigo was found to be more rapid in presence of AhdA1bA2b, even with no gene induction. On the other hand, AhdA1cA2c, AhdA1dA2d and AhdA1eA2e broadly clustered with biochemically characterized *o*-substituted benzoate dioxygenases. The recombinant strains expressing AhdA1cA2c and AhdA1dA2d along with the ET components (AhdA3 and AhdA4) could transform salicylic acid to catechol by salicylate 1-hydroxylase activity as reported in other sphingomonads [9,27,28]. These recombinant strains were also shown to transform anthranilic acid to 2-aminophenol, however, no detectable metabolite was identified when 2-chloro- or 2-iodobenzoate were used as substrates. No such activities could be detected in the recombinant strain harboring AhdA1eA2e which might be due to truncated form of AhdA1e. XylX–XylY, clustered with typical benzoate/toluate dioxygenase, when expressed along with the ET components (AhdA3 and AhdA4), showed transformation of benzoic acid and *p*-toluic acid to catechol and 4-methylcatechol, respectively. Salicylate 1-hydroxylase and benzoate/*p*-toluate dioxygenase activities were further confirmed by incubating the respective reaction mixture in the presence of another recombinant strain forming catechol 2,3-dioxygenase (XylE) furnishing yellow colored products with characteristic absorbance around 340 nm, indicating the formation of 2-hydroxymuconic semialdehyde or its methyl derivative. Biotransformed products, catechol, methylcatechol and 2-aminophenol were subsequently characterized by HPLC analyses by comparing the retention times and UV-visible spectra (obtained from diode array analysis) with those of the authentic compounds analyzed under identical conditions (data not shown).

### 2.7 Other putative ORFs in the metabolism of aromatic compounds

Among the other identified putative enzymes, dihydrodiol dehydrogenase (AhdB) and 1,2-dihydroxybenzylpyruvate aldolase (NahE) showed maximum identity of 51.73 and 62% with the biochemically characterized homologous enzymes from *Burkholderia* sp. DBT1 [29]. While 2-hydroxychromene-2-carboxylate isomerase (NahD) and dihydroxy cyclohexadiene carboxylate dehydrogenase (XylL) displayed 58.47 and 58.75% identity with the homologous enzymes from *Mycobacterium vanbaalenii* PYR-1 [30] and *Cycloclasticus* sp. P1 [31], respectively. One of the two putative ring-cleavage dioxygenases, AhdC, showed 74.16 and 73.48% identity with 1,2-dihydroxynaphthalene dioxygenase from *Rhodococcus* sp. TFB and 2,3-dihydroxybiphenyl-1,2-dioxygenase from *Rhodococcus jostii* RHA1, respectively [32,33] while the other, XylE showed 95.11 and 52.45% identity with catechol 2,3-dioxygenase from *Sphingomonas* sp. TZS-7 and *Pseudomonas* sp. CF600 respectively [34,35]. In addition, few more putative catabolic upper pathway enzymes (XylM, XylA, XylB and XylC) and lower pathway enzymes (XylF, XylG, XylJ, XylQ and XylK) involved in the metabolism of xylene and 2-hydroxymuconic semialdehyde, respectively, were identified (Table S2). Sequenced clusters also encode a putative NtrC-type regulator (AhdR), largely reported to be involved in the regulation of aromatic degradation pathway enzymes [36] and a TetR-type transcriptional regulator. However, *ahdR* in strain PNB is disrupted by insertion of a transposase. Sequenced clusters also encoded one each of pyruvate phosphate dikinase, TonB-dependent receptor protein, glutathione S-transferase, 4-hydroxythreonine-4-phosphate dehydrogenase, IS4 family transposase and three hypothetical proteins, whose role in aromatic degradation could not be determined.

### 2.8 Real-time PCR analyses

**Fig. 5:**
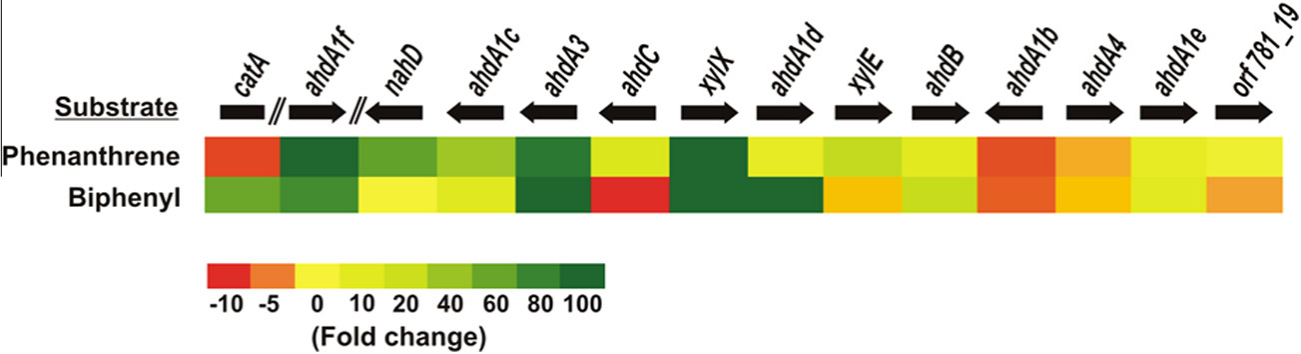
Real-time PCR analysis of genes in *Sphingobium* sp. PNB involved in the metabolism of aromatic hydrocarbons. Heat map representing expression levels of different genes, induced with phenanthrene and biphenyl. The fold change is shown in shades of red, yellow and green which indicate decreased, unchanged and increased levels of expression, respectively. Fold change denotes change in expression level of a gene in induced cells compared to the uninduced (succinate grown) cells. Double slash (//) represents gap between distantly located genes or genes present in different loci. Orientation of *catA*, identified in different loci, with respect to sequenced gene clusters is not known. (For interpretation of the references to colour in this figure legend, the reader is referred to the web version of this article.)

Results obtained from real-time PCR analyses with cDNA synthesized from the respective RNAs isolated from cells grown in presence of either phenanthrene or biphenyl are shown in Fig. 5. With the exception of *ahdA1b* and *ahdA4*, most of the genes were overexpressed in phenanthrene-grown cells as compared with succinate-grown cells. However, *ahdA1b*, *ahdA4*, *nahD*, *ahdC* or *xylE* were not overexpressed in biphenyl-grown cells. On the other hand, *catA* (GenBank: KC683533), encoding catechol 1,2-dioxygenase, was found to be upregulated in biphenyl grown cells but marginally downregulated in phenanthrene grown culture.

## 3 Discussion

The ability to degrade diverse harmful aromatic compounds by sphingomonads may be linked to the presence of evolutionarily unique enzyme system. Evolutionary relationships among several RHOs in sphingomonads have already been studied [37–41], which showed a radical divergence of their RHO genes from those of other genera, indicating a restriction in genetic exchange between sphingomonads and non-sphingomonads [3]. The presence of multiple peripheral enzymes (terminal oxygenases) and a single copy of each of the downstream degradative enzymes in the catabolic clusters are unique to strain PNB and various other sphingomonads [8,13]. Moreover, it has already been reported that the multiple RHOs from sphingomonads interact with only a single set of the corresponding ET system [3]. Effectively, the maximal activity of RHOs is shown to require the specific ET proteins, ferredoxin and ferredoxin reductase. Although the specific ET proteins can be partially replaced by endogenous *E. coli* ET proteins at the cost of reduced activity of RHOs but the role of ferredoxin is more significant than that of reductase in productive catalysis [42,43]. The ET proteins from sphingomonads are reported to be quite flexible in their redox partner interactions as together they are capable of transferring electrons to some of the oxygenase components of the RHOs from several non-sphingomonads. On the contrary, although the reductase from sphingomonads could be replaced by other reductase from non-sphingomonads, alternative ferredoxin components from non-sphingomonads failed to transfer electrons to the terminal oxygenase component of RHOs in sphingomonads [44]. Again, a single ferredoxin present in the degradative cluster of sphingomonad strain CHY-1 has been reported to have varied affinities for the different terminal oxygenases [28].

Thus, it is interesting to understand the structural nature of a single ferredoxin component capable of transferring electrons to structurally diverse terminal oxygenases in sphingomonads. In the present study, molecular modeling followed by docking analysis of ferredoxin component with the heterohexameric α_3_β_3_-type terminal oxygenase of strain PNB reflect its unique structural configuration to bind at a pronounced depression formed by two α- and two β-subunits, similar to that observed with the structurally characterized subunits from *Sphingobium yanoikuyae* B1. However, similar analysis with the corresponding proteins from *Pseudomonas putida* NCIB 9816-4 revealed striking differences, binding at the other depression formed by the two adjacent α-subunits of NDO-O_98164_ [26]. Unique structural configuration of ferredoxin in sphingomonads has also been reflected from the presence of differentially conserved amino acids, which are also involved as interface residues in protein-protein interactions. Indeed, structural information of the rest of the oxygenases in sphingomonads will help to understand the mechanism of interaction of a single ferredoxin with multiple terminal oxygenases.

Seven pairs of α- and β-subunits identified in strain PNB correspond to the analogous subunits of RHOs (*bphA1A2*_[a–f]_ and *xylXY*) in *Sphingomonas yanoikuyae* B1 [13], *Novosphingobium aromaticivorans* F199 [8] and *Sphingomonas* sp. LH128 (NCBI BioProject: PRJNA172017). Based on phylogenetic relationships and substrate preferences of α-subunits in strain PNB, transformation of putative substrate(s) and related compounds by recombinant strains, expressing individual RHO terminal oxygenases along with sole available set of constituent ET components indicate their broad substrate specificities.

The α-subunit of the terminal oxygenase corresponding to AhdA1f responsible for initial dioxygenation of a number of PAHs and polycyclic heteroaromatic hydrocarbons has been well characterized in strains, viz. LH128 [10], B1 [7] and CHY-1 [45] which are respectively 99.92, 76.71 and 76.62% identical to AhdA1f. On the other hand, AhdA1bA2b, functionally reported for the first time, is capable of transforming alkylbenzenes, such as ethylbenzene, propylbenzene, cumene and *p*-cymene, in addition to the aromatics described for AhdA1fA2f. Based on the formation of biotransformed products (Table 1), differences in regiospecificity of AhdA1fA2f and AhdA1bA2b have been noticed for a number of PAHs. AhdA1fA2f can transform phenanthrene to both phenanthrene *cis*-3,4-dihydrodiol (retention time, R_t_ 11.15 min) and phenanthrene *cis*-1,2-dihydrodiol (R_t_ 13.67 min) in contrast to the formation of phenanthrene *cis*-3,4-dihydrodiol (R_t_ 11.20 min) only with AhdA1bA2b. Phenanthrene *cis*-3,4-dihydrodiol and phenanthrene *cis*-1,2-dihydrodiol have already been reported in the phenanthrene degradation pathways via 1-hydroxy-2-naphthoic acid and 2-hydroxy-1-naphthoic acid, respectively in strain PNB [11]. Based on phylogenetic affiliation, AhdA1b uniquely clustered together with the homologous sequences from other sphingomonads and evolved from the common ancestor of largely defined PAHs and alkyl- and/or arylbenzene (which includes biphenyl) dioxygenase showing closest relationship with the biochemically characterized ethylbenzene dioxygenases from *Rhodococcus jostii* RHA1 and *Rhodococcus* sp. DK17, which are 100% identical at protein level. However, ethylbenzene dioxygenases from strain RHA1 was also reported to transform various aromatic compounds, including benzene, biphenyl, ethylbenzene and naphthalene, with the latter as preferred substrate. Functionally, AhdA1bA2b showed characteristics of both A-IIIαβ and A-IVαβ type RHOs, justifying the phylogenetic affiliation of AhdA1b within the tree.

Salicylate hydroxylase (AhdA1cA2c and AhdA1dA2d) described in strain PNB and homologous proteins reported in related sphingomonads viz. strains P2, B1 and CHY-1 [9,27,28] are the only three-component decarboxylative monooxygenases that transform salicylic acid to catechol. Apart from salicylate-1-hydroxylase, anthranilate-1-hydroxylase is reported in strain PNB, similar to that reported in CHY-1. On the other hand, XylX-XylY, as reported earlier, showed transformation of benzoic acid and *p*-toluic acid to catechol and 4-methylcatechol respectively. Thus it is believed that the presence of multiple copies of highly conserved RHO α- and β-subunits and their broad substrate specificities may likely provide strain PNB a pronounced selective advantage in the management of a wide range of aromatics present in the environment. Although phylogenetic analyses revealed the substrate preference of AhdA1aA2a towards phenanthrene and/or hetero-substituted aromatics such as dibenzothiophene and dibenzofuran but this could not be validated experimentally owing to the disrupted nature of this gene in strain PNB. Although the substrate preference of AhdA1eA2e was predicted to be salicylic acid, this particular RHO α-subunit being truncated in strain PNB, failed to show any oxygenase activity.

The presence of multiple transposons and insertion elements in strain PNB as well as in the genome of other sphingomonads strongly indicates pronounced DNA rearrangements [3,46], and suggests significant roles for them in the localization of the conserved gene clusters and establishment of the degradation pathways for various compounds [47–49]. Comparison of the genome sequences of *Sphingobium chlorophenolicum* L-1 and *Sphingobium japonicum* UT26 suggests horizontal gene transfer events in the pentachlorophenol degradation pathway [50]. The complex genetic architecture of sphingomoanads was further revealed by the presence of a number of overlapping genes (*nahD*-*ahdA1c*, *ahdA2c*-*ahdA3*, *xylX*-*xylY*-*orf183_9*, *ahdA1d*-*ahdA2d*, *xylQ*-*xylK* and *ahdA2a*-*ahdA1a*) in strain PNB, involved in aromatic degradation. The presence of overlapping genes is thought to be the result of evolutionary pressure to conserve sequence length [51–53], minimize genome size and regulate gene expression [52,54,55].

Compared to the succinate-grown cells, phenanthrene or biphenyl induced cells of strain PNB showed upregulation of many genes present in the gene clusters reported in this study. Generally, the groups of genes transcribed in the same frame were upregulated simultaneously. However, in biphenyl grown cells, *ahdC* was downregulated in spite of being in the same frame with the genes, necessary for the upper pathway of degradation of various aromatics. Again, *nahD*, encoding an isomerase essential for the degradation of phenanthrene but not for biphenyl, was found to be overexpressed in phenanthrene-grown cells but not in biphenyl-grown cells. Expression profiles of *catA* and *xylE* (Fig. 5) suggest that the central metabolite catechol is processed through *ortho* (β-ketoadipate) pathway in biphenyl and benzoic acid degradation but through *meta* (α-ketoadipate) pathway in case of phenanthrene, naphthalene and salicylic acid degradation. Interestingly, although AhdA1b, along with the corresponding β-subunit and ET components, was able to transform a large spectrum of aromatics including biphenyl and phenanthrene (Table 1), its expression was downregulated in presence of either of the substrates. Again AhdA4, which is present as a single copy in the degradation cluster and is essential for functional activity of the multiple RHOs, was not overexpressed in presence of either biphenyl or phenanthrene. Moreover, neither salicylic acid nor benzoic acid/*p*-toluic acid-grown cells were found to overexpress the majority of the degradative genes reported in this study. Thus it is believed that the expression of degradative gene cluster is more specific towards inducible substrates rather than the transformation of a range of compounds by the peripheral enzymes. Based on the prediction of regulation, it has already been suggested that multiple inducers are required for the expression of aromatic catabolic enzymes in sphingomonads [56]. Moreover, it is likely that genes, which are not overexpressed but essential for the degradation of a particular compound, must be complemented by the expression of appropriate catabolic genes present in other location in the genome. In this context, it may be mentioned that the analysis of genomes of different aromatics-degrading sphingomonads revealed the presence of multiple copies of degradative genes, in addition to those present in the aromatic-degradative clusters reported in this study [46].

As mentioned above, the regulation of genes for various aromatics degradation in sphingomonads is quite complex. The *ahdR* gene encoding a putative regulator belonging to the NtrC family was identified in close proximity to the genes coding for the catabolism of aromatic compounds. Members of this family are known to activate RNA polymerase containing the alternative sigma factor σ^54^. Homologs of *ahdR* are found in many sphingomonads having similar organization of degradative genes. Analyses of promoter region sequences of the catabolic plasmid pNL1 in strain F199 suggested that regulatory events are modulated through the interaction of BphR with σ^54^ type promoters [56]. However, its actual role in aromatic degradation cannot be ascertained as truncated versions of the gene are found not only in *Sphingomonas* sp. P2, *Novosphingobium pentaromativorans* US6-1 but also in strain PNB with the insertion of transposase (*orf26*, Fig. 1), all of which show similar degradative gene arrangement and reported to successfully mineralize phenanthrene or high molecular weight PAHs. Apart from *ahdR*, a gene (*orf781_19*) encoding TetR-type transcriptional regulator, often involved in aromatic degradation [36], is also present in the degradative cluster of strain PNB similar to that observed in strain LH128 (Fig. 1). However, the role of TetR-type regulator in the regulation of these proximal genes cannot be contemplated as the same is absent in rest of the sphingomonads compared in this study. Thus, the present study lays the groundwork for revealing the answers to the molecular basis of the underlying complex regulation of gene expression involved in the degradation of broad spectrum aromatics in sphingomonads.

## 4 Experimental procedures

### 4.1 Amplification and identification of RHO α-subunit genes from strain PNB

RHO α-subunit genes, belonging to the seven paralogous groups (annotated as *bphA1a*-*bphA1f* and *xylX* in *Novosphingobium aromaticivorans* F199, GenBank: NC_002033) were subjected to amplification from strain PNB using degenerate primers (Table S1). Primers were designed based on MSA of nucleic acid sequences obtained from various reported phenanthrene-degrading sphingomonads (Table S3). PCR amplifications were performed with a MJ Mini Gradient Thermal Cycler (Bio-Rad Laboratories, Inc.) followed by sequencing of amplified PCR products according to the manufacturer’s specifications for Taq DNA polymerase-initiated cycle sequencing reactions using fluorescent-labeled dideoxynucleotide terminators with an ABI PRISM 377 automated sequencer (Perkin-Elmer Applied Biosystems, Inc.). Sequence homology analyses were performed using both blastn and blastx programs [57], available at the NCBI (NIH, Bethesda, MD).

### 4.2 Construction and screening of genomic library

*Sphingobium* sp. PNB was grown on Luria-Bertani (LB) broth overnight at 28 °C. Genomic DNA from the strain PNB was isolated and purified according to Marmur and Doty [58] with certain modifications and improvisations, as suggested by Lambert *et al*. [59]. A genomic library was prepared in pCC2FOS Fosmid vector (Epicentre, Madison, Wisconsin) according to the manufacturer’s protocol. Briefly, the genomic DNA from strain PNB was randomly sheared to approximately 40 kb fragments and the ends were blunted using End-repair Enzyme Mix (CopyControl^TM^ HTP Fosmid Library Production Kit, Epicentre) and ligated into pCC2FOS vector. The ligated DNA was packaged with MaxPlax Lambda Packaging Extracts and transduced into EPI300-T1R Phage T1-resistant *E. coli* Plating strain followed by spreading onto LB-agar plates containing 12.5 μg ml^−1^ chloramphenicol. The resulting library was replica-plated and the clones containing RHO(s) were screened by PCR amplification using primers as described above.

### 4.3 Subcloning and sequencing

Fosmid DNA was individually isolated using the Miniprep SpinKit (Qiagen Inc., Stanford, USA) from the clones containing various RHO genes. Isolated DNA was then digested individually with PstI, SmaI, HindIII, XhoI and BglII and the DNA fragments ranging from 2–8 kb were subcloned into pBluescript SK(−) and transformed into *E.coli* XL1-Blue cells. The transformants were plated onto LB-ampicillin plate containing 20 μg ml^−1^ of 5-bromo-4-chloro-3-indolyl-beta-D-galactopyranoside (X-gal) and 0.1 mM IPTG. The plates were incubated overnight at 37 °C. The transformants were then screened by PCR for the presence of various RHO α-subunit genes. For sequencing, the recombinant plasmids were isolated and subjected to DNA sequencing using M13 forward and reverse primers, followed by primer walking in both directions. Gaps between genes located in close proximity were bridged by conventional primer walking method. Both DNA sequencing and sequence homology analyses were carried out as described above.

### 4.4 Phylogenetic analyses

Homologous RHO α-subunits (Table S3), ferredoxin (Table S4) and reductase (Table S5) protein sequences were identified with blastp program [57] against the non-redundant (NR) database at NCBI using each RHO α-subunit paralog, AhdA3 and AhdA4 from strain PNB as query sequences. ClustalX v1.81 [60] was used to generate individual MSA of RHO α-subunits, ferredoxin and reductase protein sequences obtained from strain PNB and those of the corresponding homologous sequences from other sphingomonads and non-sphingomonads followed by manual adjustment, wherever necessary. Phylogenetic trees were constructed by neighbor-joining (NJ) method from distance data using the NJ algorithm implemented in ClustalX. The trees were visualized and manipulated either using the program Tree Explorer v2.12 [61] or using iTOL: Interactive Tree Of Life, an online phylogenetic tree viewer and Tree Of Life resource [62].

### 4.5 *In silico* analysis

The homology models of monomers of oxygenase α-subunit (AhdA1f), β-subunit (AhdA2f) and ferredoxin (AhdA3) were generated using the software MODELLER 9v7 [63] with the respective oxygenase components (PDB: 2GBX:A; 2GBX:B) and ferredoxin (PDB: 2I7F:A) of the biphenyl 2,3-dioxygenase from *Sphingobium yanoikuyae* B1. The models were checked using PROCHECK [64], VERIFY3D [65], VADAR [66] and PSIPRED [67]. For docking experiments, structures of the terminal oxygenase and ferredoxin components of naphthalene dioxygenase from *Pseudomonas putida* NCIB 9816-4 (NDO-O_98164_ and NDO-F_98164_) and those of biphenyl dioxygenase from *Sphingobium yanoikuyae* B1 (BDO-O_B1_ and BDO-F_B1_) were downloaded from the PDB. The monomeric structures of oxygenase α-subunit (AhdA1f) and β-subunit (AhdA2f) from strain PNB, modeled above, were used to construct the heterohexameric form of the enzyme (AHD-O_PNB_) so as to dock with the corresponding ferredoxin (AHD-F_PNB_). GRAMM-X [68] was used to predict and assess the interactions between NDO-O_98164_ and NDO-F_98164_, BDO-O_B1_ and BDO-F_B1_ as well as AHD-O_PNB_ and AHD-F_PNB_. The program performs a rigid-body docking using Fast FT methods by applying smoothed Lennard-Jones potential to find protein complexes with the highest surface complementarity. From each docking, 50 most probable predictions (in order from most to least favorable) based on geometry, hydrophobicity and electrostatic complementarity of the molecular surface were considered for further analyses. The interface residues in the docked complex were predicted using ProFace server [69].

### 4.6 Construction of plasmids for protein overexpression

Primers were designed (Table S7) to amplify *ahdA1bA2b*, *ahdA1cA2c*, *ahdA1dA2d*, *ahdA1eA2e, ahdA1fA2f*, *xylXY*, *ahdA3*, *ahdA4* and *xylE* genes from genomic DNA of strain PNB. The pET28a vector (Novagen, Madison, WI) was used for cloning the PCR amplicons *ahdA1bA2b*, *ahdA1cA2c*, *ahdA1dA2d*, *ahdA1eA2e, ahdA1fA2f*, *xylXY* and *xylE* genes while pCDF-1b vector for that of *ahdA3* followed by *ahdA4* into the chimeric plasmid. Next, the recombinant vectors were transformed into *E. coli* BL21(DE3) for expression analysis.

### 4.7 Overexpression of oxygenases and *in vivo* assays

Different *E. coli* BL21(DE3) cells containing one of the RHOs, ferredoxin and reductase genes were grown overnight in 5 ml LB medium in presence of appropriate antibiotics. These cultures were used to inoculate 100 ml LB medium (0.1% v/v) and were incubated at 37 °C until an OD_600_ of 0.5 was reached. After inducing the cultures with IPTG (0.5 mM), the cells were further incubated overnight at 25 °C. For *in vivo* assays, cells were centrifuged, washed and resuspended to an OD_600_ of 2.0 in M9 medium [70] supplemented with 0.2% glucose. Cells overexpressing various components of RHO were incubated overnight at 25 °C with 400 μM of each of the test substrate dissolved in 2 ml of silicone oil.

### 4.8 Chemical analyses

After incubation, the resting cell cultures were centrifuged (8,000×*g*, 10 min) and the supernatants were adjusted to pH 7.0 and extracted thrice with an equal volume of ethyl acetate. The combined extracts were dried over anhydrous sodium sulfate, evaporated under reduced pressure and finally resuspended in 100 μl *N,N*-dimethylformamide (DMF). Then, 100 μl of *n*-butylboronic acid solution (500 μg of *n*-butylboronic acid dissolved in 1 ml of DMF) was added, and the mixture was heated at 70 °C for 15 min to form the NBB derivatives. The reaction mixture thus obtained was diluted 15 fold with cyclohexane and analyzed by GC-MS using a Varian model 3800 (Varian Inc., California, USA) with a Saturn 2200 mass spectrometer equipped with a 30 m × 0.25 mm (0.25 μm film thickness) DB5 MS capillary column (Agilent Technologies, California, USA). The inlet temperature was kept at 285 °C while the transfer line temperature was kept at 270 °C. The temperature program gave a 2 min hold at 80 °C, an increase to 260 °C at 18 °C min^−1^, followed by hold for 6 min at 260 °C, further increase to 285 °C at 4 °C min^−1^ and a 11 min hold at 285 °C. The injection volume was 1 μl, and the carrier gas was helium (1 ml min^−1^). The mass spectrometer was operated at an electron ionization energy of 70 eV and dihydrodiols were detected by selected ion monitoring by using the calculated mass of the NBB derivative. On the other hand, low molecular weight polar metabolites were resolved by HPLC using a Shimadzu model LC20-AT pump system equipped with a diode array model SIL-M20A detector and a C_18_ reversed-phase column attached to a model SIL-20A autosampler. The biotransformed products were eluted using a programmed gradient solvent system at a flow rate of 1.0 ml min^−1^ and detected at 254 nm along with diode array analysis. The mobile phase, consisting of methanol and water containing 1% (v/v) acetic acid, was a 45 min linear gradient from 50% (v/v) to 95% (v/v) aqueous methanol with hold at 95% (v/v) aqueous methanol for 10 min followed by 95% (v/v) to 50% (v/v) aqueous methanol over 5 min.

### 4.9 RNA isolation, cDNA preparation and real-time PCR analysis

Total RNA was isolated using TRIzol (Invitrogen, Carlsbad, CA) from mid-exponential phase cultures of strain PNB grown individually on phenanthrene, biphenyl or succinate as sole carbon sources. Residual DNA was removed by additional treatment with RNase-free DNase I (Thermo Scientific, Waltham, MA). Subsequently, cDNA was prepared with RevertAid reverse transcriptase (Thermo Scientific) and Random Hexamer Primer (Thermo Scientific), according to the manufacturer’s instructions. To quantitatively estimate expression of genes involved in degradation of different aromatics, real-time PCR was performed in an ABI 7500 real-time PCR system (Applied Biosystems, California, USA) with various sets of primers (Table S8) using SYBR Green mix and cDNAs prepared from different set of cells. Relative changes in mRNA expression of various genes were compared with succinate as control, normalized to 16S rRNA, and quantified by the 2^−ΔΔCt^ method [71]. Mean values were obtained from triplicate experiments.

### 4.10 Nucleotide sequence accession numbers

The nucleotide sequences described in this study were deposited into GenBank database under the accession numbers GenBank: KF483792, GenBank: KF483793 and GenBank: KF483794.

## Figures and Tables

**Table 1:** GC-MS data for the oxidation products showing aromatic hydrocarbon selectivity of AhdA1bA2b and AhdA1fA2f from *Sphingobium* sp. PNB as expressed in *E. coli*.^a^

Substrate^b^	Product	Molecular mass of NBB derivative	R_t_ (min)		Relative activity (%)^c^	
			AhdA1bA2b	AhdA1fA2f	AhdA1bA2b	AhdA1fA2f
Naphthalene	Naphthalene dihydrodiol	228	10.25	10.23	46.4	100
Biphenyl	Biphenyl dihydrodiol	254	11.45	11.44	8.5	54.1
Phenanthrene	Phenanthrene dihydrodiol 1 Phenanthrene dihydrodiol 2	278 278	11.20 ND	11.15 13.67	12.5 ND	28.1 22.4
Anthracene	Anthracene dihydrodiol	278	14.12	14.03	14.7	26.4
Acenaphthene	Acenaphthene dihydrodiol	254	14.11	14.10	2.6	5.2
Benz[a] anthracene	Benz[a]anthracene dihydrodiol 1 Benz[a]anthracene dihydrodiol 2	328 328	ND 18.39	18.24 18.39	ND 0.2	0.7 1.7
Benzo[a] pyrene	Benzo[a]pyrene dihydrodiol 1 Benzo[a]pyrene dihydrodiol 2	352 352	20.78 ND	20.77 20.93	0.9 ND	1.2 1.7
Ethylbenzene	Ethylbenzene dihydrodiol	206	8.44	ND	43.7	ND
Propylbenzene	Propylbenzene dihydrodiol	220	11.00	ND	22.9	ND
Cumene	Cumene dihydrodiol	220	10.15	ND	6.05	ND
*p*-Cymene	*p*-Cymene dihydrodiol	234	12.45	ND	8.40	ND

a Abbreviations: R_t_, retention time; ND, not detected.

b Pyrene and fluoranthene did not give any detectable product.

c Calculated from the GC-MS-selected ion-monitoring peak areas of the NBB derivatives of the products formed after 16 h of incubation and expressed as percentages of relative activity (with respect to the maximum obtained with naphthalene as substrate for AhdA1fA2f). The values are averages of two separate determinations.

## Appendix A Supplementary data

Supplementary data Supplementary data contains Tables and Figures.

## References

[1] Takeuchi M., Hamana K., Hiraishi A. (2001). Proposal of the genus *Sphingomonas sensu strictu* and three new genera, *Sphingobium*, *Novosphingobium* and *Sphingopyxis*, on the basis of phylogenetic and chemotaxonomic analyses. Int. J. Syst. Evol. Microbiol..

[2] Gibson D.T., Parales R.E. (2000). Aromatic hydrocarbon dioxygenases in environmental biotechnology. Curr. Opin. Biotechnol..

[3] Stolz A. (2009). Molecular characteristics of xenobiotic-degrading sphingomonads. Appl. Microbiol. Biotechnol..

[4] Parales R.E., Resnick S.M., Ramos J.-L., Levesque R.C. (2006). Aromatic ring hydroxylating dioxygenase. Pseudomonas.

[5] Butler C.S., Mason J.R. (1997). Structure–function analysis of the bacterial aromatic ring-hydroxylating dioxygenases. Adv. Microb. Physiol..

[6] Chakraborty J., Ghosal D., Dutta A., Dutta T.K. (2012). An insight into the origin and functional evolution of bacterial aromatic ring-hydroxylating dioxygenases. J. Biomol. Struct. Dyn..

[7] Ni Chadhain S.M., Moritz E.M., Kim E., Zylstra G.J. (2007). Identification, cloning, and characterization of a multicomponent biphenyl dioxygenase from *Sphingobium yanoikuyae* strain B1. J. Ind. Microbiol. Biotechnol..

[8] Romine M.F., Stillwell L.C., Wong K.K., Thurston S.J., Sisk E.C., Sensen C., Gaasterland T., Fredrickson J.K., Saffer J.D. (1999). Complete sequence of a 184-kilobase catabolic plasmid from *Sphingomonas aromaticivorans* F199. J. Bacteriol..

[9] Pinyakong O., Habe H., Yoshida T., Nojiri H., Omori T. (2003). Identification of three novel salicylate 1-hydroxylases involved in the phenanthrene degradation of *Sphingobium* sp. strain P2. Biochem. Biophys. Res. Commun..

[10] Schuler L., Jouanneau Y., Ní Chadhain S.M., Meyer C., Pouli M., Zylstra G.J., Hols P., Agathos S.N. (2009). Characterization of a ring-hydroxylating dioxygenase from phenanthrene-degrading *Sphingomonas* sp. strain LH128 able to oxidize benz[*a*]anthracene. Appl. Microbiol. Biotechnol..

[11] Roy M., Khara P., Dutta T.K. (2012). *meta*-cleavage of hydroxynaphthoic acids in the degradation of phenanthrene by *Sphingobium* sp. strain PNB. Microbiology.

[12] Roy M., Khara P., Basu S., Dutta T.K. (2013). Catabolic versatility of *Sphingobium* sp. strain PNB capable of degrading structurally diverse aromatic compounds. J. Biorem. Biodegrad..

[13] Zylstra G.J., Kim E. (1997). Aromatic hydrocarbon degradation by *Sphingomonas yanoikuyae* B1. J. Ind. Microbiol. Biotechnol..

[14] Jiang H., Parales R.E., Lynch N.A., Gibson D.T. (1996). Site-directed mutagenesis of conserved amino acids in the alpha subunit of toluene dioxygenase: potential mononuclear non-heme iron coordination sites. J. Bacteriol..

[15] Kim S., Chun J., Bae K., Kim Y. (2000). Polyphasic assignment of an aromatic-degrading *Pseudomonas* sp., strain DJ77, in the genus *Sphingomonas* as *Sphingomonas chungbukensis* sp. nov. Int. J. Syst. Evol. Microbiol..

[16] Willison J.C. (2004). Isolation and characterization of a novel sphingomonad capable of growth with chrysene as sole carbon and energy source. FEMS Microbiol. Lett..

[17] Suen W.C., Gibson D.T. (1993). Isolation and preliminary characterization of the subunits of the terminal component of naphthalene dioxygenase from *Pseudomonas putida* NCIB 9816-4. J. Bacteriol..

[18] Parales R.E. (2003). The role of active-site residues in naphthalene dioxygenase. J. Ind. Microbiol. Biotechnol..

[19] Jakoncic J., Jouanneau Y., Meyer C., Stojanoff V. (2007). The crystal structure of the ring-hydroxylating dioxygenase from *Sphingomonas* CHY-1. FEBS J..

[20] Iwasaki T., Miyauchi K., Masai E., Fukuda M. (2006). Multiple-subunit genes of the aromatic-ring-hydroxylating dioxygenase play an active role in biphenyl and polychlorinated biphenyl degradation in *Rhodococcus* sp. strain RHA1. Appl. Environ. Microbiol..

[21] Harayama S., Rekik M., Bairoch A., Neidle E.L., Ornston L.N. (1991). Potential DNA slippage structures acquired during evolutionary divergence of *Acinetobacter calcoaceticus* chromosomal *benABC* and *Pseudomonas putida* TOL pWW0 plasmid *xylXYZ*, genes encoding benzoate dioxygenases. J. Bacteriol..

[22] Jencova V., Strnad H., Chodora Z., Ulbrich P., Vlcek C., Hickey W.J., Paces V. (2008). Nucleotide sequence, organization and characterization of the (halo)aromatic acid catabolic plasmid pA81 from *Achromobacter xylosoxidans* A8. Res. Microbiol..

[23] Nierman W.C., DeShazer D., Kim H.S., Tettelin H., Nelson K.E., Feldblyum T., Ulrich R.L., Ronning C.M., Brinkac L.M., Daugherty S.C. (2004). Structural flexibility in the *Burkholderia mallei* genome. Proc. Natl. Acad. Sci. USA.

[24] Rieske J.S., MacLennan D.H., Coleman R. (1964). Isolation and properties of an iron-protein from the (reduced coenzyme Q)-cytochrome C reductase complex of the respiratory chain. Biochem. Biophys. Res. Commun..

[25] Page C.C., Moser C.C., Chen X., Dutton P.L. (1999). Natural engineering principles of electron tunnelling in biological oxidation–reduction. Nature.

[26] Ashikawa Y., Fujimoto Z., Noguchi H., Habe H., Omori T., Yamane H., Nojiri H. (2006). Electron transfer complex formation between oxygenase and ferredoxin components in Rieske nonheme iron oxygenase system. Structure.

[27] Cho O., Choi K.Y., Zylstra G.J., Kim Y.S., Kim S.K., Lee J.H., Sohn H.Y., Kwon G.S., Kim Y.M., Kim E. (2005). Catabolic role of a three-component salicylate oxygenase from *Sphingomonas yanoikuyae* B1 in polycyclic aromatic hydrocarbon degradation. Biochem. Biophys. Res. Commun..

[28] Jouanneau Y., Micoud J., Meyer C. (2007). Purification and characterization of a three-component salicylate 1-hydroxylase from *Sphingomonas* sp. strain CHY-1. Appl. Environ. Microbiol..

[29] Di Gregorio S., Zocca C., Sidler S., Toffanin A., Lizzari D., Vallini G. (2004). Identification of two new sets of genes for dibenzothiophene transformation in *Burkholderia* sp. DBT1. Biodegradation.

[30] Kweon O., Kim S.J., Holland R.D., Chen H., Kim D.W., Gao Y., Yu L.R., Baek S., Baek D.H., Ahn H. (2011). Polycyclic aromatic hydrocarbon metabolic network in *Mycobacterium vanbaalenii* PYR-1. J. Bacteriol..

[31] Lai Q., Li W., Wang B., Yu Z., Shao Z. (2012). Complete genome sequence of the pyrene-degrading bacterium *Cycloclasticus* sp. strain P1. J. Bacteriol..

[32] Tomás-Gallardo L., Santero E., Camafeita E., Calvo E., Schlömann M., Floriano B. (2009). Molecular and biochemical characterization of the tetralin degradation pathway in *Rhodococcus* sp. strain TFB. Microb. Biotechnol..

[33] Takeda H., Shimodaira J., Yukawa K., Hara N., Kasai D., Miyauchi K., Masai E., Fukuda M. (2010). Dual two-component regulatory systems are involved in aromatic compound degradation in a polychlorinated-biphenyl degrader, *Rhodococcus jostii* RHA1. J. Bacteriol..

[34] Lu J., Nomura N., Nakajima-Kambe T., Nakahara T. (2000). Cloning and expression of genes encoding *meta*-cleavage enzymes from 4,6-dimethyldibenzothiophene-degrading *Sphingomonas* strain TZS-7. Biochim. Biophys. Acta.

[35] Bartilson M., Shingler V. (1989). Nucleotide sequence and expression of the catechol 2,3-dioxygenase-encoding gene of phenol-catabolizing *Pseudomonas* CF600. Gene.

[36] Tropel D., van der Meer J.R. (2004). Bacterial transcriptional regulators for degradation pathways of aromatic compounds. Microbiol. Mol. Biol. Rev..

[37] Williams P.A., Sayers J.R. (1994). The evolution of pathways for aromatic hydrocarbon oxidation in *Pseudomonas*. Biodegradation.

[38] Werlen C., Kohler H.P., van der Meer J.R. (1996). The broad substrate chlorobenzene dioxygenase and *cis*-chlorobenzene dihydrodiol dehydrogenase of *Pseudomonas* sp. strain P51 are linked evolutionarily to the enzymes for benzene and toluene degradation. J. Bid. Chem..

[39] Bosch R., García-Valdés E., Moore E.R. (1999). Genetic characterization and evolutionary implications of a chromosomally encoded naphthalene-degradation upper pathway from *Pseudomonas stutzeri* AN10. Gene.

[40] Chablain P.A., Zgoda A.L., Sarde C.O., Truffaut N. (2001). Genetic and molecular organization of the alkylbenzene catabolism operon in the psychrotrophic strain *Pseudomonas putida* 01G3. Appl. Environ. Microbiol..

[41] Eby D.M., Beharry Z.M., Coulter E.D., Kurtz D.M., Neidle E.L. (2001). Characterization and evolution of anthranilate 1,2-dioxygenase from *Acinetobacter* sp. strain ADP1. J. Bacteriol..

[42] Saito A., Iwabuchi T., Harayama S. (2000). A novel phenanthrene dioxygenase from *Nocardioides* sp. strain KP7: expression in *Escherichia coli*. J. Bacteriol..

[43] Demanèche S., Meyer C., Micoud J., Louwagie M., Willison J.C., Jouanneau Y. (2004). Identification and functional analysis of two aromatic-ring-hydroxylating dioxygenases from a *Sphingomonas* strain that degrades various polycyclic aromatic hydrocarbons. Appl. Environ. Microbiol..

[44] Yu C.L., Liu W., Ferraro D.J., Brown E.N., Parales J.V., Ramaswamy S., Zylstra G.J., Gibson D.T., Parales R.E. (2007). Purification, characterization, and crystallization of the components of a biphenyl dioxygenase system from *Sphingobium yanoikuyae* B1. J. Ind. Microbiol. Biotechnol..

[45] Jouanneau Y., Meyer C., Jakoncic J., Stojanoff V., Gaillard J. (2006). Characterization of a naphthalene dioxygenase endowed with an exceptionally broad substrate specificity toward polycyclic aromatic hydrocarbons. Biochemistry.

[46] Aylward F.O., McDonald B.R., Adams S.M., Valenzuela A., Schmidt R.A., Goodwin L.A., Woyke T., Currie C.R., Suen G., Poulsen M. (2013). Comparison of 26 sphingomonad genomes reveals diverse environmental adaptations and biodegradative capabilities. Appl. Environ. Microbiol..

[47] Dogra C.V., Raina V., Pal R., Suar M., Lal S., Gartemann H.-H., Holliger C., van der Meer J.R., Lal R. (2004). Organization of *lin* genes and IS6100 among different species of hexachlorocyclohexane degrading *Sphingomonas paucimobilis*: evidence for horizontal gene transfer. J. Bacteriol..

[48] Müller T.A., Byrde S.M., Werlen C., van der Meer J.R., Kohler H.-P.E. (2004). Genetic analysis of phenoxyalkanoic acid degradation in *Sphingomonas herbicidovorans* MH. Appl. Environ. Microbiol..

[49] Thiel M., Kaschabek S., Gröning J., Mau M., Schlömann M. (2005). Two unusual chlorocatechol catabolic gene clusters in *Sphingomonas* sp. TFD44. Arch. Microbiol..

[50] Copley S.D., Rokicki J., Turner P., Daligault H., Nolan M., Land M. (2012). The whole genome sequence of *Sphingobium chlorophenolicum* L-1: insights into the evolution of the pentachlorophenol degradation pathway. Genome Biol. Evol..

[51] Miyata T., Yasunaga T. (1978). Evolution of overlapping genes. Nature.

[52] Krakauer D.C. (2000). Stability and evolution of overlapping genes. Evolution.

[53] Scherbakov D.V., Garber M.B. (2000). Overlapping genes in bacterial and phage genomes. Mol. Biol..

[54] Keese P.K., Gibbs A. (1992). Origins of genes: "big-bang" or continuous creation?. Proc. Natl. Acad. Sci. USA.

[55] Johnson Z.I., Chisholm S.W. (2004). Properties of overlapping genes are conserved across microbial genomes. Genome Res..

[56] Romine M.F., Fredrickson J.K., Li S.-M.W. (1999). Induction of aromatic catabolic activity in *Sphingomonas aromaticivorans* strain F199. J. Ind. Microbiol. Biotechnol..

[57] Altschul S.F., Gish W., Miller W., Myers E.W., Lipman D.J. (1990). Basic local alignment search tool. J. Mol. Biol..

[58] Marmur J., Doty P. (1961). Thermal renaturation of deoxyribonucleic acids. J. Mol. Biol..

[59] Lambert L.H., Cox T., Mitchell K., Rossello-Mora R.A., Del Cueto C., Dodge D.E., Orkand P., Cano R.J. (1998). *Staphylococcus succinus* sp. nov., isolated from Dominican amber. Int. J. Syst. Bacteriol..

[60] Thompson J.D., Gilson T.J., Plewniak F., Jeanmougin F., Higgins D.G. (1997). The ClustalX windows interface: flexible strategies for multiple sequence alignment aided by quality analysis tools. Nucleic Acids Res..

[61] Tamura K., Dudley J., Nei M., Kumar S. (2007). MEGA4: molecular evolutionary genetics analysis (MEGA) software version 4.0. Mol. Biol. Evol..

[62] Letunic I., Bork P. (2011). Interactive tree of life v2: online annotation and display of phylogenetic trees made easy. Nucleic Acids Res..

[63] Sali A., Blundell T.L. (1993). Comparative protein modelling by satisfaction of spatial restraints. J. Mol. Biol..

[64] Laskowski R.A., MacArthur M.W., Moss D.S., Thornton J.M. (1993). Procheck: a program to check the stereochemical quality of protein structures. J. Appl. Crystallogr..

[65] Luthy R., Bowie J.U., Eisenberg D. (1992). Assessment of protein models with three-dimensional profiles. Nature.

[66] Willard L., Ranjan A., Zhang H., Monzavi H., Boyko R.F., Sykes B.D., Wishart D.S. (2003). VADAR: a web server for quantitative evaluation of protein structure quality. Nucleic Acids Res..

[67] McGuffin L.J., Bryson K., Jones D.T. (2000). The PSIPRED protein structure prediction server. Bioinformatics.

[68] Tovchigrechko A., Vakser I.A. (2006). GRAMM-X public web server for protein–protein docking. Nucleic Acids Res..

[69] Saha R.P., Bahadur R.P., Pal A., Mandal S., Chakrabarti P. (2006). ProFace: a server for the analysis of the physicochemical features of protein-protein interfaces. BMC Struct. Biol..

[70] Sambrook J., Fritsch E.F., Maniatis T. (1989). Molecular Cloning: A Laboratory Manual.

[71] Schmittgen T.D., Livak K.J. (2008). Analyzing real-time PCR data by the comparative C_T_ method. Nat. Protoc..

