# Supplementary material for: Functional characterization of diverse ring-hydroxylating oxygenases and induction of complex aromatic catabolic gene clusters in Sphingobium sp. PNB
Source: FEBS Open Bio. 2014 Mar 7;4:290–300. doi: 10.1016/j.fob.2014.03.001 (PMC4048848; doi:10.1016/j.fob.2014.03.001)
Supplement: Supplementary data — contains Tables and Figures. [file mmc1.doc]

**SUPPORTING INFORMATION:**

**Functional characterization of diverse ring-hydroxylating oxygenases and induction of complex aromatic catabolic gene clusters in *Sphingobium* sp. PNB**

Pratick Khara, Madhumita Roy, Joydeep Chakraborty, Debajyoti Ghosal, Tapan K. Dutta

Department of Microbiology, Bose Institute, P-1/12 C.I.T. Scheme VII M, Kolkata 700054, India.

**CONTENTS:**

- Table S1
- Table S2
- Table S3
- Table S4
- Table S5
- Table S6
- Table S7
- Table S8
- Fig. S1

**Table S1.** Primers used to amplify RHO α-subunit genes from strain PNB.

| Gene^a^ | Primers | Sequence (5' - 3') |
| --- | --- | --- |
| *bphA1a* | A1a_F | CCTATTTCGGCGAACTCG |
|  | A1a_R | GAARTAGCGCTGRTTGTG |
| *bphA1b* | A1b_F | ACGKATCGGYCGYGARCCG |
|  | A1b_R | GTCGCGSGTTTCGGCCAC |
| *bphA1c* | A1c_F | GGWYGCCGGGTTCCSTAC |
|  | A1c_R | RTGGTCGGCATCCTCGGC |
| *bphA1d* | A1d_F | CCRACKGCGGTSGAYTAC |
|  | A1d_R | GCATAGCCGAAATAGGTC |
| *bphA1e* | A1e_F | AAYGARGTTCCSAAGGCG |
|  | A1e_R | ATATTCCCARCGGGGMAG |
| *bphA1f* | A1f_F | TGCCAYGATCCCGAAGCG |
|  | A1f_R | VGCCCAATCKGGBGCATC |
| *xylX* | X_F | GGYMGGATTGCCGAYTTGC |
|  | X_R | GGCMACCGCCGGGCTGC |

^a^ Homologous gene corresponding to *Novosphingobium aromaticivorans* F199.

**Table S2. ORFs identified from different fosmid clones bearing aromatic hydrocarbon catabolic genes in *Sphingobium* sp. PNB.**

| ORF | Frame | Homologous gene product (BlastX) | | | | | Putative gene in strain PNB |
| --- | --- | --- | --- | --- | --- | --- | --- |
|  |  | Functional description | Matching protein | % Identity | Organism | GenBank  Accession No. |  |
| Fosmid clone: FC-31 | | | | | | | |
| Orf31_1 | - | Hypothetical protein belonging to the drug/metabolite transporter (DMT) superfamily | - | 95.28 | *Novosphingobium aromaticivorans*  F199 | NP_049063 | *orf31_1* |
| Orf31_2 | + | PAH dioxygenase large (α) subunit | NahA1f | 99.92 | *Sphingomonas* sp. LH128 | EJU15002 | *ahdA1f* |
| Orf31_3 | + | PAH dioxygenase small (β) subunit | NahA2f | 100 | *Sphingomonas* sp. LH128 | EJU15003 | *ahdA2f* |
| Orf31_4 | + | Alcohol dehydrogenase | XylB | 99.90 | *Sphingomonas* sp. LH128 | EJU15004 | *xylB* |
| Orf31_5 | + | Pyruvate phosphate dikinase | PpdK | 100 | *Sphingomonas* sp. LH128 | EJU15005 | *orf31_5* |
| Fosmid clone: FC-183 | | | | | | | |
| Orf183_1 | - | TonB-dependent receptor | - | 92.91 | *Novosphingobium aromaticivorans* DSM 12444 | ABP64115 | *orf183_1* |
| Orf183_2 | - | 2-Hydroxychromene-2-carboxylate isomerase | NahD | 100 | *Sphingomonas* sp. LH128 | EJU14546 | *nahD* |
| Orf183_3 | - | Salicylate 1-hydroxylase large (α) subunit | BphA1c | 100 | *Sphingomonas* sp. LH128 | EJU14547 | *ahdA1c* |
| Orf183_4 | - | Salicylate 1-hydroxylase small (β) subunit | BphA2c | 100 | *Sphingomonas* sp. LH128 | EJU14548 | *ahdA2c* |
| Orf183_5 | - | Rieske-type ferredoxin | BphA3 | 100 | *Sphingomonas* sp. LH128 | EJU14549 | *ahdA3* |
| Orf183_6 | - | Dihydroxy PAH/biphenyl dioxygenase | BphC | 100 | *Sphingomonas* sp. LH128 | EJU14550 | *ahdC* |
| Orf183_7 | + | Toluate/benzoate dioxygenase large (α) subunit | xylX | 100 | *Sphingomonas* sp. LH128 | EJU14551 | *xylX* |
| Orf183_8 | + | Toluate/benzoate dioxygenase small (β) subunit | XylY | 100 | *Sphingomonas* sp. LH128 | EJU14552 | *xylY* |
| Orf183_9 | + | 4-Hydroxythreonine-4-phosphate dehydrogenase | - | 100 | *Novosphingobium aromaticivorans*  F199 | AAD04003 | *orf183_9* |
| Orf183_10 | + | Aromatic oxygenase large (α) subunit | BphA1d | 100 | *Sphingomonas* sp. LH128 | EJU14554 | *ahdA1d* |
| Orf183_11 | + | Aromatic oxygenase small (β)subunit | BphA2d | 100 | *Sphingomonas* sp. LH128 | EJU14555 | *ahdA2d* |
| Orf183_12 | + | Glutathione S-transferase | BphK | 100 | *Sphingomonas* sp. LH128 | EJU14556 | *bphK* |
| Orf183_13 | + | 2-Hydroxymuconic semialdehyde hydrolase | - | 100 | *Sphingobium chungbukense*  DJ77 | AAC45091 | *xylF* |
| Orf183_14 | + | Catechol 2,3-dioxygenase | XylE | 100 | *Sphingobium chungbukense*  DJ77 | AAB41537 | *xylE* |
| Orf183_15 | + | Hypothetical protein | - | 100 | *Sphingobium chungbukense*  DJ77 | AAB66312 | *orf183_15* |
| Orf183_16 | + | 2-Hydroxymuconic semialdehyde dehydrogenase | XylG | 100 | *Sphingomonas* sp. LH128 | EJU14560 | *xylG* |
| Orf183_17 | + | 2-Hydroxypent-2, 4-dienoate hydratase | XylJ | 100 | *Sphingomonas* sp. LH128 | EJU14561 | *xylJ* |
| Orf183_18 | + | Acetaldehyde dehydrogenase | XylQ | 100 | *Sphingomonas* sp. LH128 | EJU14562 | *xylQ* |
| Orf183_19 | + | 4-Hyroxy-2-oxovalerate/4-Hydroxy-2-oxopentanoic acid aldolase | XylK | 100 | *Sphingomonas* sp. LH128 | EJU14563 | *xylK* |
| Fosmid clone: FC-781 | | | | | | | |
| Orf781_1 | - | Benzaldehyde dehydrogenase | XylC | 99.59 | *Sphingomonas* sp. LH128 | EJU14567 | *xylC* |
| Orf781_2 | - | Dihydrodiol dehydrogenase | BphB | 99.22 | *Sphingomonas* sp. LH128 | EJU14568 | *ahdB* |
| Orf781_3 | - | Xylene monooxygenase electron transfer subunit | XylA | 100 | *Sphingomonas* sp. LH128 | EJU14569 | *xylA* |
| Orf781_4 | - | Xylene monooxygenase hydroxylase subunit | XylM | 100 | *Sphingomonas* sp. LH128 | EJU14570 | *xylM* |
| Orf781_5 | - | Hypothetical protein | - | 100 | *Sphingomonas* sp. LH128 | EJU14100 | *orf781_5* |
| Orf781_6 | - | Aromatic oxygenase small (β)subunit | BphA2b | 100 | *Sphingomonas* sp. LH128 | EJU14099 | *ahdA2b* |
| Orf781_7 | - | Aromatic oxygenase large (α)subunit | BphA1b | 100 | *Sphingomonas* sp. LH128 | EJU14098 | *ahdA1b* |
| Orf781_8 | - | Aromatic oxygenase small (β)subunit | BphA2a | 96 | *Novosphingobium aromaticivorans*  F199 | AAD03981 | *ahdA2a* |
| Orf781_9 | - | Aromatic oxygenase large (α)subunit | BphA1a | 100 (199 residues at C-terminus of BphA1a) | *Sphingomonas* sp. LH128 | EJU10799 | *ahdA1a* |
| Orf781_10 | - | Transposase Tn3 family protein | - | 100 | *Sphingobium chlorophenolicum* L-1 | YP_004556195 | *orf781_10* |
| Orf781_11 | + | Resolvase | TnpR | 100 | *Sphingomonas* sp. KA1 | YP_717948 | *orf781_11* |
| Orf781_9 | - | Aromatic oxygenase large (α)subunit | BphA1a | 100 (251 residues from N-terminus of BphA1a) | *Sphingomonas* sp. LH128 | EJU10799 | *ahdA1a* |
| Orf781_12 | + | Regulator of aromatic degradative pathways | BphR | 95.07 (528 residues from N-terminus of BphR) | *Novosphingobium aromaticivorans*  F199 | AAD03979 | *ahdR* |
| Orf781_13 | - | Transposase of ISsp1 | Tnp | 99.56 | *Sphingobium japonicum* UT26S | YP_003544147 | *orf781_13* |
| Orf781_12 | + | Regulator of aromatic degradative pathways | BphR | 94.73 (19 residues at C-terminus of BphR) | *Novosphingobium aromaticivorans*  F199 | AAD03979 | *ahdR* |
| Orf781_14 | + | Ferredoxin reductase subunit of aromatic oxygenase | BphA4 | 100 | *Sphingomonas* sp. LH128 | EJU12838 | *ahdA4* |
| Orf781_15 | - | Dihydroxy cyclohexadiene carboxylate  dehydrogenase | XylL | 98.47 | *Novosphingobium aromaticivorans*  F199 | AAD03977 | *xylL* |
| Orf781_16 | + | 1,2-Dihydroxybenzylpyruvate aldolase | NahE | 99.69 | *Sphingomonas* sp. LH128 | EJU12841 | *nahE* |
| Orf781_17 | + | Aromatic oxygenase large (α) subunit | BphA1e | deleted | *Sphingomonas* sp. LH128 | EJU12842 | *ahdA1e* |
| Orf781_18 | + | Aromatic oxygenase small (β) subunit | BphA2e | 100 | *Sphingomonas* sp. LH128 | EJU12843 | *ahdA2e* |
| Orf781_19 | + | TetR family transcriptional regulator | - | 100 | *Sphingomonas* sp. LH128 | EJU12844 | *orf781_19* |
| Orf781_20 | - | IS4 family transposase | - | 100 | *Sphingomonas* sp. LH128 | EJU12845 | *orf781_20* |

### Table S3. Oxygenases α-subunit sequences from different organisms used for phylogenetic analysis.

| Organism | Oxygenase large subunit | |
| --- | --- | --- |
|  | Protein | GenBank  Accession No. |
| *Acinetobacter* sp. ADP1 | AntA  BenA | AAC34813  AAC46436 |
| *Alcaligenes faecalis* AFK2 | PhnAc | BAA76323 |
| *Burkholderia cepacia* DB01 | AntAc | AAO83639 |
| *Burkholderia cepacia* R34 | DntAc | AAL50021 |
| *Burkholderia mallei* ATCC 23344 | OhbB | AAU46052 |
| *Burkholderia* sp. 2CBS | CbdA | CAA55681 |
| *Burkholderia* sp. DBT1 | DbtAc | AAK62353 |
| *Burkholderia* sp. PS12 | TecA1 | AAC46390 |
| *Burkholderia* sp. RASC | DntAc | AAB09766 |
| *Burkholderi*a sp. RP007 | PhnAc | AAD09872 |
| *Burkholderia xenovorans* LB400 | CmtAb | YP_557484 |
| *Comamonas* sp. JS764 | NbzAc | AAL76202 |
| *Cupriavidus oxalaticus* A5 | BphA1 | CAD61140 |
| *Cycloclasticus* sp. A5 | PhnA1 | BAC81541 |
| *Mycobacterium vanbaalenii* PYR-1 | NidA3  PhtAa | AAY85176  AAQ91914 |
| *Nocardioides* sp. KP7 | PhdA | BAA94708 |
| *Novosphingobium aromaticivorans* F199 | BphA1f  BphA1e  BphA1a  BphA1b  BphA1d  XylX  BphA1c | NP_049062  NP_049179  NP_049184  NP_049186  NP_049206  NP_049209  NP_049213 |
| *Novosphingobium pentaromativorans* US6-1 | AhdA1c  XylX  BphA1d  AhdA1e  PhnA1a | EHJ58008  EHJ58012  EHJ58015  EHJ58035  EHJ58049 |
| *Polaromonas naphthalenevorans* CJ2 | NagG  NagAc | AAZ93385  AAZ93388 |
| *Pseudomonas aeruginosa* JB2 | HybB | AAC69484 |
| *Pseudomonas aeruginosa* PA01 | XylX | AAG05906 |
| *Pseudomonas aeruginosa* PAO1 | AntA | AAG05900 |
| *Pseudomonas fluorescens* IP01 | CumA1 | BAA07074 |
| *Pseudomonas pseudoalcaligenes*  KF707 | BphA1 | AAA25743 |
| *Pseudomonas putida* | XylX | AAA26047 |
| *Pseudomonas putida* F1 | TodC1  CmtAb | AAA26005  AAB62285 |
| *Pseudomonas putida* G7 | NahAc | AAA25902 |
| *Pseudomonas putida* NCIB- 98164 | NahAc | AAO64274 |
| *Pseudomonas putida* O1G3 | EbdAa | CAB99196 |
| *Pseudomonas putida* OUS82 | PahAc | BAA20391 |
| *Pseudomonas resinovorans* CA10 | AntA | BAB32747 |
| *Pseudomonas* sp. JS42 | NtdAc | AAB40383 |
| *Pseudomonas stutzeri* AN10 | NahAc | AAD02136 |
| *Ralstonia* sp. U2 | NagG  NagAc | AAD12607  AAD12610 |
| *Rhodococcus* *jostii* RHA1 | BphA1  BenA  EtbAa1  PadAa1 | BAA06868  BAB70698  YP_708484  YP_707370 |
| *Rhodococcus* sp. 19070 | BopX | AAK58903 |
| *Rhodococcus* sp. DK17 | AkbA1a | AAR90139 |
| *Rhodococcus* sp. I24 | NidA | AAD25395 |
| *Rhodococcus* sp. P200 | NarAa | AAR05114 |
| *Rhodopseudomonas palustris* No.7 | PsbAb | BAA82116 |
| *Sphingobium yanoikuyae* B1 | BphA1c  XylX  BphA1d  BphA1b  BphA1a  BphA1e  BphA1f | ABM79781  ABM79785  ABM79788  ABM79807  ABM79809  ABM79814  ABM91740 |
| *Sphingomonas* sp. CB3 | CarAa | AAC38616 |
| *Sphingomonas* sp. CHY-1 | PhnA1a  PhnA1b | CAG17576  CAG17582 |
| *Sphingomonas* sp. KA1 | AntAc  CarAaI | YP_717961  YP_717981 |
| *Sphingomonas* sp. LH128 | BphA1a  BphA1e  BphA1b  BphA1c  XylX  BphA1d  NahA1f | EJU10799  EJU12842  EJU14098  EJU14547  EJU14551  EJU14554  EJU15002 |
| *Sphingomonas* sp. P2 | AhdA1c  XylX  AhdA1d  AhdA1b  AhdA1a  AhdA1e | BAC65426  BAC65430  BAC65433  BAC65446  BAC65448  BAC65453 |
| *Sphingomonas* sp. RW1 | DxnA1 | CAA51365 |
| *Terrabacter* sp. DBF63 | PhtA1 | BAC54156 |
| *Terrabacter* sp. YK3 | DfdA1 | BAC06602 |

### Table S4. Ferredoxin [2Fe-2S] sequences from different organisms used for phylogenetic analysis.

| Organisms | Ferredoxin | |
| --- | --- | --- |
|  | Protein | GenBank  Accession No. |
| *Alcaligenes faecalis* AFK2 | PhnAb | BAA76320 |
| *Burkholderia cepacia* DBO1 | AntAb | AAO83641 |
| *Burkholderia cepacia* R34 | DntAb | AAL50022 |
| *Burkholderia mallei* ATCC 23344 | OhbC | AAU46050 |
| *Burkholderia* sp. RASC | DntAb | AAB09765 |
| *Burkholderia xenovorans* LB400 | BphF | AAB63428 |
| *Burkholderia xenovorans* LB400 | CmtAd | YP_557488 |
| *Comamonas* sp. JS765 | NbzAb | AAL76201 |
| *Comamonas testosteroni* TK102 | BphA3 | BAC01054 |
| *Cycloclasticus* sp. A5 | PhnA3 | BAC81547 |
| *Novosphingobium aromaticivorans* F199 | BphA3 | NP_049211 |
| *Novosphingobium pentaromativorans* US6-1 | BphA3 | EHJ58010 |
| *Polaromonas naphthalenivorans* CJ2 | NagAb | AAZ93387 |
| *Pseudomonas fluorescens* IPO1 | CumA3 | BAA07077 |
| *Pseudomonas pseudoalcaligenes* KF707 | BphA3 | AAA25746 |
| *Pseudomonas putida* | BnzA3 | AAA25737 |
| *Pseudomonas putida* F1 | CmtAd | AAB62289 |
| *Pseudomonas putida* G7 | NahAb | AAA25901 |
| *Pseudomonas putida* NCIB 9816-4 | NahAb | AAA25905 |
| *Pseudomonas putida* O1G3 | EbdAc | CAB99198 |
| *Pseudomonas putida* OUS82 | PahAb | BAA20390 |
| *Pseudomonas* sp. JS42 | NtdAb | AAB40382 |
| *Pseudomonas stutzeri* AN10 | NahAb | AAD02135 |
| *Ralstonia* sp. U2 | NagAb | AAD12609 |
| *Rhodococcus jostii* RHA1 | EtbAc | YP_708496 |
| *Rhodococcus jostii* RHA1 | BphA3 | BAA06870 |
| *Sphingobium yanoikuyae* B1 | BphA3 | ABM79783 |
| *Sphingomonas* sp. CHY-1 | PhnA3 | CAG17580 |
| *Sphingomonas* sp. LH128 | BphA3 | EJU14549 |
| *Sphingomonas* sp. P2 | AhdA3 | BAC65428 |
| *Terrabacter* sp. YK3 | DfdA3 | BAC06604 |

### Table S5. Glutathione-reductase (GR)-type ferredoxin reductase sequences from different organisms used for phylogenetic analysis.

| Organisms | Ferredoxin | |
| --- | --- | --- |
|  | Protein | GenBank  Accession No. |
| *Burkholderia cepacia* DBO1 | AntAa | AAO83642 |
| *Burkholderia mallei* ATCC 23344 | OhbD | AAU46049 |
| *Burkholderia xenovorans* LB400 | BphG | AAB63429 |
| *Burkholderia xenovorans* LB400 | CmtAa | YP_557483 |
| *Nocardioides* sp. KP7 | PhdD | BAA94714 |
| *Novosphingobium aromaticivorans* F199 | BphA4 | NP_049182 |
| *Novosphingobium pentaromativorans* US6-1 | AhdA4 | EHJ58032 |
| *Pseudomonas fluorescens* IPO1 | CumA4 | BAA07078 |
| *Pseudomonas pseudoalcaligenes* KF707 | BphA4 | AAA25747 |
| *Pseudomonas putida* | BnzA4 | AAA25738 |
| *Pseudomonas putida* F1 | CmtAa | AAB62284 |
| *Pseudomonas putida* O1G3 | EbdAd | CAB99199 |
| *Rhodococcus jostii* RHA1 | EtbAd | YP_708476 |
| *Rhodococcus jostii* RHA1 | BphA4 | BAA06871 |
| *Rhodococcus jostii* RHA1 | PadAd1 | YP_707365 |
| *Sphingobium yanoikuyae* B1 | BphA4 | ABM79811 |
| *Sphingomonas* sp. CB3 | CarAd | AAC38619 |
| *Sphingomonas* sp. LH128 | BphA4 | EJU12838 |
| *Sphingomonas* sp. P2 | AhdA4 | BAC65450 |
| *Terrabacter* sp. DBF63 | PhtA4 | BAC54161 |
| *Terrabacter* sp. YK3 | DfdA4 | BAC06605 |

### Table S6. Interface residues between the terminal oxygenase α-subunit and ferredoxin of structurally characterized ring-hydroxylating oxygenase systems compared to the modeled system that of strain PNB.

| Interacting proteins^a^ | Terminal oxygenase α-subunit | | Ferredoxin |
| --- | --- | --- | --- |
|  | Rieske domain | Catalytic domain |  |
| NDO-O_98164_ and NDO-F_98164_ | Ser73, Ile74, Arg75, Arg82, Ser127, Leu128, Asn129, Lys131, Cys132, Leu133, Glu137, Val138, Ala139, Arg140, Val141, Gln154 | Ile7, Ser10, Glu11, Ser12, Leu14, Ser15, Lys17, Asp382, Leu384, Asn386, Phe389, Lys445 | Thr1, Val2, Trp4, Asp41, Leu43, Cys44, Thr45, His46, Lys76, Ala77, Leu78, Cys79, Ala80, Pro81, Val82, Tht83, Gln84, Asn85, Ile86, Lys87, Pro90, Val91, Asp101, Leu102, Ser103 |
| BDO-O_B1_ and BDO-F_B1_ | Lys49, Asn94, Ala95, Val99, Val106, Asp115, Val116, Pro117 | Trp210, Ala213, Ala227, Gly228, Asn229, Arg230, Ala231, Asp232 | Tyr22, Glu24, Asp54, Tyr56, Glu63, Cys64, Pro65, Phe66, His67, Gly68, Ala80, Phe81 |
| AHD-O_PNB_ and AHD-F_PNB_ | Ser94, Ala95, Lys96, Ala97, Phe98, Val99, Gly104, Val106, Val114, Asp115, Pro117 | Trp210, Thr211, Ala213, Ala214, Gln217, Met218, Ser227, Gly228, Arg230 | Leu44, Cys45, Thr46, His47, Gly48, Asn49, Asp54, Gly55, Thr56, Glu63, Cys64, Pro65, Phe66, His67, Gly68, Phe81, Pro82, Val85 |

^a^ Abbreviations: NDO-O_98164_ and NDO-F_98164_, terminal oxygenase and ferredoxin components of naphthalene dioxygenase from *Pseudomonas putida* NCIB 9816-4; BDO-O_B1_ and BDO-F_B1_, terminal oxygenase and ferredoxin components of biphenyl dioxygenase from *Sphingobium yanoikuyae* B1; AHD-O_PNB_ and AHD-F_PNB_, terminal oxygenase and ferredoxin components of ring-hydroxylating oxygenase from *Sphingobium* sp. PNB.

**Table S7.** Primers used in this study for overexpression of different genes ^a,b^.

| Gene | Primers | | Sequence (5' - 3') | Restriction site introduced |
| --- | --- | --- | --- | --- |
| *ahdA1bA2b* | ahdAb_F | GGAATTCCATATGCGCTTCGAACGG | | NdeI |
|  | ahdAb_R | CCCAAGCTTCTAAACGTAGATGTTGAGGTTCTTG | | HindIII |
| *ahdA1cA2c* | ahdAc_F | GGAATTCCATATGGGCCTTCCCACCC | | NdeI |
|  | ahdAc_R | CGGGATCCTCATGCCGGTTCCACC | | BamHI |
| *ahdA1dA2d* | ahdAd_F | GGAATTCCATATGAACGCCGAAACCATG | | NdeI |
|  | ahdAd_R | CCCAAGCTTTCAGATCGGGGTCGCC | | HindIII |
| *ahdA1fA2f* | ahdAf_F | GGAATTCCATATGAATGGATCGTCGGCAC | | NdeI |
|  | ahdAf_R | CCCAAGCTTTTATGCGAAGAAATAGAGATTCTTGTC | | HindIII |
| *xylXY* | xylX_F | GGAATTCCATATGGAACTCAATGATAAAATCGGC | | NdeI |
|  | xylX_R | CCCAAGCTTACCCGCTCAGAGGCTG | | HindIII |
| *ahdA1eA2e* | ahdAe_F | GGAATTCCATATGGCTGACGCGACCTTG | | NdeI |
|  | ahdAe_R | CCCAAGCTTTCATATCGGGTAGACAAGATATCGC | | HindIII |
| *ahdA3* | ahdA3_F | GGGGTACCATGTCGAACCAATTGCG | | KpnI |
|  | ahdA3_R | AACTGCAGGGAAGGCCCATCAGG | | PstI |
| *ahdA4* | ahdA4_F | AACTGCAGTTGGGAGAATCGCATGAAATC | | PstI |
|  | ahdA4_R | CCGCTCGAGCTAACCAGCCTGCTTGAGGATATC | | XhoI |
| *xylE* | xylE_F | GGAATTCCATATGGCTTTGACTGGTGTAATTCG | | NdeI |
|  | xylE_R | CCCAAGCTTTCAGGTGTTCACCGTCATG | | HindIII |

^a^ Restriction sites are shown as underlined.

^b^ Ribosome binding site (RBS) is shown as double underlined.

**Table S8.** Primers used for real-time PCR analysis of genes from *Sphingobium* sp. PNB.

| Gene | Primers | Sequence (5' - 3') |
| --- | --- | --- |
| 16S rRNA | 16S rRNA_ RT_F | GTAGGCGGCGATTTAAGTC |
|  | 16S rRNA_ RT_R | ACAATCGTCCAGTGAGCC |
| *xylE* | xylE _ RT_F | CGTGGATATAGCCAGTTCG |
|  | xylE _ RT_R | TACCGTCTTCGGGATAGC |
| *ahdA1f* | ahdA1f_ RT_F | ACAAGGGCTTCATTTTCG |
|  | ahdA1f_ RT_R | CTCGGTCGGGACTTTCC |
| *ahdA1b* | ahdA1b_ RT_F | GCCTCAAGAACGTCGC |
|  | ahdA1b_ RT_R | ACCGACTTGATGCAGCC |
| *ahdA1c* | ahdA1c_ RT_F | CTGAGACAGTTACGCACCG |
|  | ahdA1c_ RT_R | AAGCTTCCAATTCCCGC |
| *ahdA1d* | ahdA1d_ RT_F | AGCATTCGCTGGAGCG |
|  | ahdA1d_ RT_R | ATAGCCCAGAACCTTGACC |
| *ahdA1e* | ahdA1e_ RT_F | AATGGCATCCGGTTGC |
|  | ahdA1e_ RT_R | TTGTTACCCATTACCGCAG |
| *xylX* | xylX _ RT_F | AAGTCCGGCGATGTCG |
|  | xylX _ RT_R | ATGCTCTCATCGGTGACG |
| *ahdA3* | ahdA3_ RT_F | GCAGTATATAATGTTGATGGCG |
|  | ahdA3_ RT_R | ATCGCCACTTGGCAGG |
| *ahdA4* | ahdA4_ RT_F | AACGTGCATTACCTGCG |
|  | ahdA4_ RT_R | TCATAGGCGCGGCAAG |
| *ahdB* | ahdB_ RT_F | GATGTCCGCAATTATGTCG |
|  | ahdB_ RT_R | CTTTACGTTGACGCCGAAG |
| *ahdC* | ahdC_ RT_F | ATGACCTGGCCTACATGG |
|  | ahdC_ RT_R | GATCTCGGTCGGATTGCC |
| *nahD* | nahD_ RT_F | GTCTTGCCCAAGATCAAGG |
|  | nahD_ RT_R | CGGTCACAAATGCCTCG |
| *orf781_19* | orf781_19_ RT_F | GAGAGTGGCTACGATAATGTCAC |
|  | orf781_19_ RT_R | TTATACTACTACATTCGGAAATATCGAG |
| *catA* | catA_ RT_F | GCTTCCATTCGAAGATGC |
|  | catA_ RT_R | CTGCGTCGTCAGCGTG |


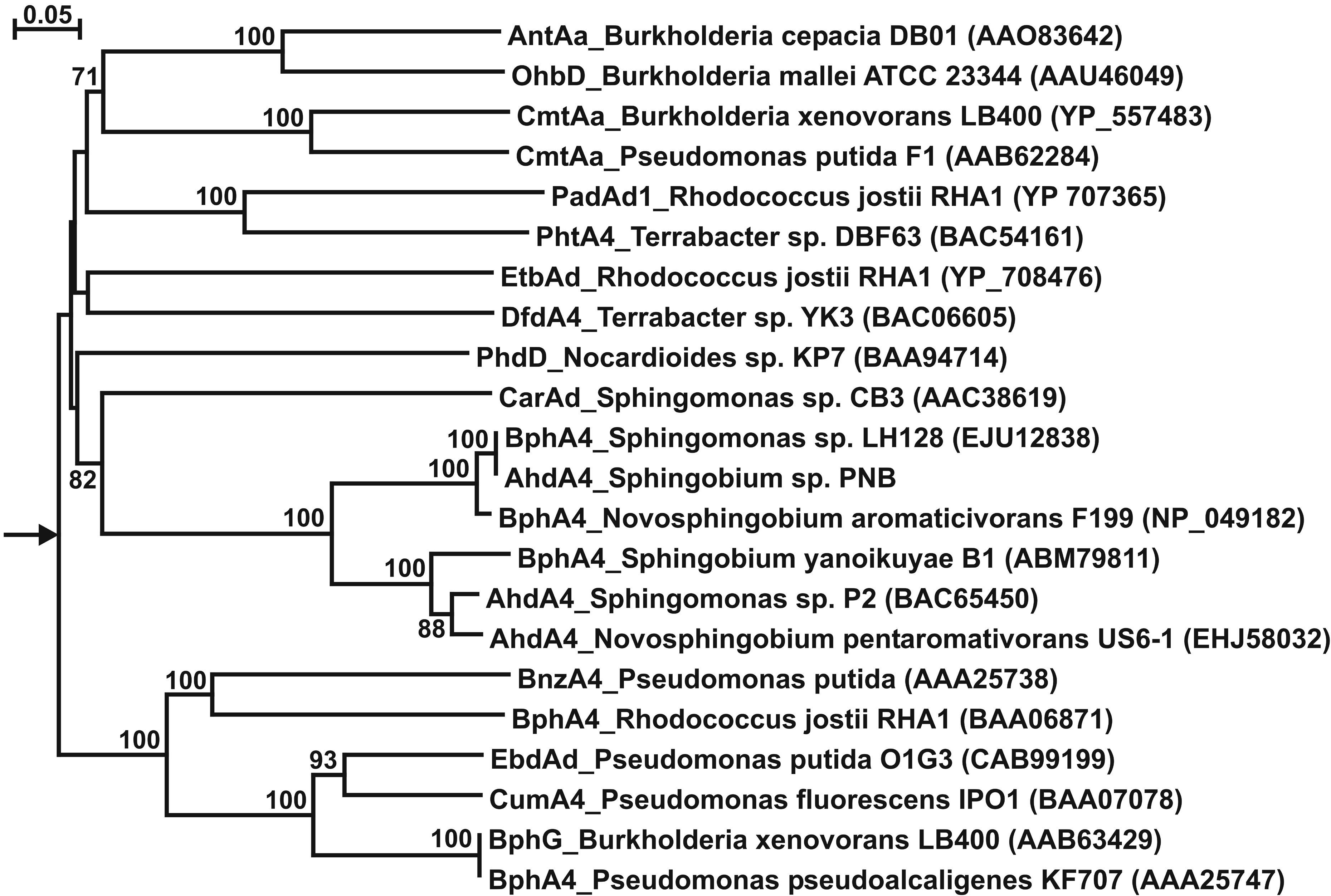


**Fig. S1.** Neighbor-joining tree of Glutathione Reductase (GR) - type ferredoxin reductases from different well studied xenobiotic degrading bacteria. Values at each node indicate level of bootstrap support based on 100 resampled datasets while bootstrap values below 50% are not shown. An unrelated reductase (NahAa) from *Pseudomonas putida* NCIB-981641 (GenBank: AAA25904) was used as outgroup and position of the root has been indicated by an arrow. Bar represents 0.05 substitutions per amino acid.
